# Phototransduction in retinal cones: Analysis of parameter importance

**DOI:** 10.1371/journal.pone.0258721

**Published:** 2021-10-28

**Authors:** Colin Klaus, Giovanni Caruso, Vsevolod V. Gurevich, Heidi E. Hamm, Clint L. Makino, Emmanuele DiBenedetto

**Affiliations:** 1 The Mathematical Biosciences Institute, The Ohio State University, Columbus, Ohio, United States of America; 2 CNR, Ist. Tecnologie Applicate ai Beni Culturali, Rome, Italy; 3 Department of Pharmacology, Vanderbilt University Medical Center, Nashville, TN, United States of America; 4 Department of Physiology and Biophysics, Boston University School of Medicine, Boston, MA, United States of America; 5 Department of Mathematics, Vanderbilt University, Nashville, TN, United States of America; University Zürich, SWITZERLAND

## Abstract

In daylight, cone photoreceptors in the retina are responsible for the bulk of visual perception, yet compared to rods, far less is known quantitatively about their biochemistry. This is partly because it is hard to isolate and purify cone proteins. The issue is also complicated by the synergistic interaction of these parameters in producing systems biology outputs, such as photoresponse. Using a 3-D resolved, finite element model of cone outer segments, here we conducted a study of parameter significance using global sensitivity analysis, by *Sobol indices*, which was contextualized within the uncertainty surrounding these parameters in the available literature. The analysis showed that a subset of the parameters influencing the circulating dark current, such as the turnover rate of cGMP in the dark, may be most influential for variance with experimental flash response, while the shut-off rates of photoexcited rhodopsin and phosphodiesterase also exerted sizable effect. The activation rate of transducin by rhodopsin and the light-induced hydrolysis rate of cGMP exerted measurable effects as well but were estimated as relatively less significant. The results of this study depend on experimental ranges currently described in the literature and should be revised as these become better established. To that end, these findings may be used to prioritize parameters for measurement in future investigations.

## Introduction

Diurnal vertebrates are mostly active in fairly high light levels, when visual perception is dominated by cone photoreceptors, which are significantly less light sensitive than rods [1]. This is particularly true for modern humans using artificial lights to enable cone vision. In fact, out of more than 90,000,000 photoreceptors in the human retina, approximately 100,000 cones concentrated in the virtually rod-free fovea are used for the tasks requiring high spatial acuity, such as reading [1]. The structure of rods, in which a narrow connecting cilium is located between the outer segment containing visual transduction machinery and the rest of the cell, made preparation of fairly pure rod outer segments feasible many decades ago. This was performed simply by breaking the cell at the connecting cilium and then using density gradients to separate outer segments from other retinal components (reviewed in [2]). A high abundance of rods, constituting more than 90% of all photoreceptors in the retinas of most model species, resulted in high yields of rod-specific proteins, which allowed their biochemical characterization. Extensive sets of rod phototransduction parameters are now available for several species, including mouse [3], which are very similar to human. Hence, most biochemically detailed models of visual transduction described rods [4–10]. Few model animals have a cone-dominated retina; ground squirrel and tree shrew are notable exceptions [11, 12]. As a consequence, the preparation of relatively pure cone outer segments suitable for biochemical characterization of transduction components is often not possible although progress in cone purification techniques has been made, for example, with carp [13, 14]. While the general structure of the signaling cascade and its shutoff mechanisms are similar in rods and cones, cones use many distinct phototransduction proteins including critical components of the cascade: photopigment, G-protein transducin, effector phosphodiesterase, cyclic nucleotide gated channel (reviewed in [15]). Thus, one cannot rely on known rod parameters to model cone responses.

At present the characterization of cone-specific proteins is woefully incomplete, and there is no model species for which all the values necessary for quantitative modeling have been experimentally determined. Here we analyzed the impact of variation of individual parameters on the predictions of our space-resolved model of cone signaling [16]. Parameter ranges were coarsely suggested by interspecies measurements and, in the face of such uncertainty, we used *global sensitivity analysis* (GSA) to identify parameters that are most influential as measured by variance in model output and therefore first priority for future investigations. It should be emphasized that parameter influence was measured with respect to literature uncertainty which may not yet coincide with that of biological significance. In contrast to *local sensitivity analysis*, which depends on a particular choice of fitting parameters, GSA measures parameter importance when these vary over prescribed ranges. To the authors’ knowledge this is the first time GSA, by Sobol indices [17, 18], has been applied to phototransduction. Our mathematical model has proved particularly useful for predicting the functional behavior of rod photoreceptors [6, 8, 19–21]. Now GSA with a revised version of this model, adapted to cones [16], has evidenced that a subset of parameters which determine the dark-adapted state of the photoreceptor are the most influential, over the presented uncertainty ranges, for reproducing experimental cone flash responses. The most significant parameter was found to be the turnover rate of cGMP in the dark, *β*_*dark*_. The second most significant was a newly derived parameter that quantifies how nearly the photoreceptor is biochemically tuned towards the *impossibility* of a dark-adapted state, *a*_*min*_. This latter quantity has strong physical meaning and originates from the need for balance between cGMP synthesis by guanylyl cyclase and its hydrolysis by PDE in the dark, concurrently with the required balance between Ca^2+^ influx-efflux. In particular, sigmoidal Hill and Michaelis-Menten expressions create the possibility for maximal and minimal synthesis or hydrolysis rates (similarly for Ca^2+^ influx-efflux rates) to overwhelm the other if biochemical parameters are not properly constrained (See Eqs 9 and 10). While this finding is retrospectively intuitive, this may be the first time this constraint has been presented as potentially significant for phototransduction so that the biological range of *a*_*min*_ should be better quantified. Additional parameters which also affect the dark current and were found to be significant were the saturated exchanger current, $J_{ex}^{sat}$, and the maximum synthesis rate of cGMP by GCAP-activated guanylyl cyclase, *α*_*max*_. The shut-off rates of light-activated rhodopsin, *k*_*R*_, and phosphodiesterase, *k*_*E*_, were also significant and influential. The influence of the activation rate of transducin by photoexcited rhodopsin, *ν*_*RG*_, and the hydrolytic efficiency of the activated PDE dimer, *k*_*cat*_/*K*_*m*_, upon model variance with experiment was also appreciable but comparatively less.

## Materials and methods

Initial ranges for model parameters were based on values reported for several different species. While working within a single animal model is highly preferred, there is no complete, experimentally determined parameter set in the literature for any one species. To mitigate this situation, ranges were chosen to contain values that reproduced trends in experimental flash response without violating known parameter constraints. This selection was performed by stochastic optimization and a Metropolis-Hastings Markov Chain Monte Carlo (MCMC) random walk [22–24] over parameter space for a stationary distribution whose modes minimized root-mean-square (rms) error. Next GSA was performed to weight parameters by their relative importance. This was technically performed by the method of Sobol indices [17, 18, 25, 26]. In particular, this method demonstrated which parameters contributed the most to variance, when they were varied over their prescribed uncertainty ranges, with the examined flash response. For completeness, local sensitivity analysis is also reported.

### Proposing first ranges for parameter values of the cone outer segment

#### Geometry

Table 1 reports the geometric parameters for the mouse cone outer segment with experimental ranges from [27]. Some features of the sliver, the cytoplasmic volume that surrounds the closed section of disks and is encased by plasma membrane, were not known for mouse, so values were taken from striped bass [28] and frog [29].

**Table 1:** Geometric parameters for the mouse cone outer segment (COS).

	Unit	Description	Range	Species
*R*_*b*_	*μM*	Radius of COS base	[0.585, 0.75]	Mouse
*R*_*t*_	*μM*	Radius of COS tip	[0.38, 0.42]	Mouse
*H*	*μM*	Length of COS	[12.7, 14.1]	Mouse
*ϵ*_0_	*nm*	Disc thickness	[16.4, 17.2]	Mouse
*ν*	−	Ratio between interdiscal space and disc thickness	[0.61, 0.71]	Mouse
*σ*	−	Ratio between the disc thickness and sliver thickness	∼ 1	Striped bass
*n*	−	Number of discs	[370, 516]	Mouse
*ω*_0_	*rad*	Open margin angle for sliver	∼ *π*	Frog

#### G-protein/effector cascade

Parameters for disc membrane proteins, with their experimental ranges from the literature, were collected in Table 2. The volumic concentrations of [R]_*vol*_ = 3 *mM*, [G]_*vol*_ = 0.21 *mM*, and [PDE]_*vol*_ = 15 *μM* were reported for frog and carp in [30]. In [31] it was measured that [G]_*vol*_ in carp cone was 0.6x that in rod. These values were converted into surface densities through multiplication by the volume-to-surface conversion factor $\eta =\frac{1}{2}\nu \epsilon_{0}$ [6, 19]. For the geometric parameters in Table 1, *η* = 5.5 *nm*. The surface density of PDE on a cone disc differs considerably from the rod range of [500 *μM*^−2^, 1000 *μM*^−2^]. For *k*_*R*_, estimates for mouse rod were given [3]. The parameter range for *k*_*E*_ was based on the mouse rod value [3] and the observation that *k*_*E*_ for cones is ∼ 2.3x the mouse rod value [32]. This range is similar to the value *k*_*E*_ = 18.5 *s*^−1^ obtained by numerical fit and reported for striped bass [33], while in carp a GTP hydrolysis rate as much as ∼25x higher than rod was reported [31]. This higher estimate led to an alternative upper bound of *k*_*E*_ ∼ 150 *s*^−1^.

**Table 2:** G-protein and effector-related parameters.

	Unit	Description	Range	Species
[R]_*σ*_	*μM* ^−2^	Surface density of R	∼ 10000	Carp
[G]_*σ*_	*μM* ^−2^	Surface density of G	∼ 700	Carp
[PDE]_*σ*_	*μM* ^−2^	Surface density of PDE	∼ 50	Carp
*k*_*R*_	*s* ^−1^	Rate constant for inactivation of R*	[6.7, 12.5]	Mouse rod
*k*_*E*_	*s* ^−1^	Rate constant for inactivation of PDE*	[11.5, 16.1]	Mouse

R, rhodopsin; R*, active rhodopsin; G, transducin; PDE*, active PDE. PDE was modeled as having two subunits that could (de)activate independently.

#### Catalytic activity

Table 3 collected the activation and hydrolysis parameters used in the model. These parameters inform the model through the equations below. As before, $\eta =\frac{1}{2}\nu \epsilon_{0}$ is the volume-surface conversion factor.

$$\begin{array}{cc} {\frac{\partial \left[\text{cG}\right]}{\partial t}}_{|\text{dark}\;\text{hydrolysis}} & =\beta_{dark}{\left[\text{cG}\right]}_{dark}=\frac{k_{\sigma ;hyd}{\left[\text{PDE}\right]}_{\sigma }}{\eta }{\left[\text{cG}\right]}_{dark} \end{array}$$ (1)

$$\begin{array}{cc} {\frac{\partial \left[\text{cG}\right]}{\partial t}}_{|\text{light}\;\text{hydrolysis}} & =k_{\sigma ;hyd}^{*}{\left[{\text{PDE}}^{*}\right]}_{\sigma }\left[\text{cG}\right] \end{array}$$ (2)

$$\begin{array}{cc} k_{\sigma ;hyd}^{*} & =\frac{k_{\text{cat}}/K_{m}}{N_{Av}B_{cG}} \end{array}$$ (3)

$$\begin{array}{cc} {\frac{\partial [{\text{G}}^{*}]}{\partial t}}_{|\text{activation}} & =\nu_{RG}(\frac{{\left[\text{G}\right]}_{\sigma }-{\left[{\text{G}}^{*}\right]}_{\sigma }-2{\left[{\text{PDE}}^{*}\right]}_{\sigma }}{{\left[\text{G}\right]}_{\sigma }}){\left[{\text{R}}^{*}\right]}_{\sigma } \end{array}$$ (4)

$$\begin{array}{cc} {\frac{\partial [{\text{PDE}}^{*}]}{\partial t}}_{|\text{activation}} & =k_{GE}({\left[\text{PDE}\right]}_{\sigma }-{\left[{\text{PDE}}^{*}\right]}_{\sigma }){\left[{\text{G}}^{*}\right]}_{\sigma }. \end{array}$$ (5)

**Table 3:** Activation and hydrolysis parameters.

	Unit	Description	Range	Species
*B*_*cG*_	−	Buffering power of cytoplasm for cGMP	[1, 2]	Mouse rod
*β*_*dark*_	*s* ^−1^	Rate of cGMP hydrolysis by dark-activated PDE	[54, 846]	Carp
$k_{\sigma ;hyd}^{*}$	*μM*^3^ *s*^−1^	Surface hydrolysis rate of cGMP by PDE*	[0.75, 1.37]	Mouse rod
*k*_cat_/*K*_*m*_	*μM*^−1^ *s*^−1^	Hydrolytic efficiency of PDE* dimer	∼ 500	Frog rod
*ν*_*RG*_	*s* ^−1^	Rate of G* formation per R*	[33.0, 125.0]	Striped bass, Carp
*k*_*GE*_	*μm*^2^ *s*^−1^	Coupling coefficient for PDE* formation by G*	∼ 0.1	Carp

The ranges of *B*_*cG*_ and $k_{\sigma ;hyd}^{*}$ for mouse rod are given above [3]. The parameter value for *k*_cat_/*K*_*m*_ used by [33] in the analysis of striped bass cone was derived from measurements from frog rod [34]. *N*_Av_ is Avogadro’s number. The PDE dark activity was reported as [0.3%, 4.7%] of the maximal PDE activity, 18 cGMP/R*/*s*, in carp cone [14]. The dark rate of cGMP hydrolysis by PDE, *β*_*dark*_, was estimated by multiplying these factors by the concentration of R* in carp, [R*] = 3 *mM* [30], and then setting this resulting value to *β*_*dark*_ [*cG*]_*dark*_. The value [cG]_*dark*_ = 3 *μM* was also taken (see Table 5 for a discussion of [cG]_*dark*_).

The activation rate *ν*_*RG*_ was experimentally measured for carp in [31, 35] as *ν*_*RG*_ ≈ 33 *s*^−1^. Values for the rate of PDE activation by R*, estimated from modeling of striped bass and goldfish cones [33, 36, 37], were considered in order to estimate the range for *ν*_*RG*_. Finally, the effectiveness of transducin in carp cone to activate PDE was reported as one-tenth of its effectiveness in rod [14, 30]. This led to the estimate *k*_*GE*_ = 0.1 *μm*^2^ *s*^−1^ since in amphibian rod this value was reported as unity [38, 39].

#### Guanylyl cyclase activity

Parameters for guanylyl cyclase activity with their reported experimental ranges were given in Table 4. These parameters inform the model through the equation

$$\begin{array}{cc} {\frac{\left[\text{cG}\right]}{\partial t}}_{|\text{cyclase}} & =\alpha_{min}+\left(\alpha_{max}-\alpha_{min}\right)\frac{K_{cyc}^{m_{cyc}}}{K_{cyc}^{m_{cyc}}+{\left[{\text{Ca}}^{2+}\right]}^{m_{cyc}}}. \end{array}$$ (6)

**Table 4:** Guanylyl cyclase (GC) activity parameters. *α*_*max*_ is the maximum cGMP synthesis rate in the absence of Ca^2+^ and *α*_*min*_ is the synthesis rate at saturating Ca^2+^ concentration. These activities were measured in the absence of bicarbonate.

	Unit	Description	Range	Species
*α*_*max*_	*μMs* ^−1^	Maximum rate of cGMP synthesis	[111, 255]	Striped bass, Carp
*α*_*max*_/*α*_*min*_	−	cGMP synthesis at low relative to high [Ca^2+^]	∼ 2	Carp
*K*_*cyc*_	*nM*	Half-saturating [Ca^2+^] for GC activity	[130, 140]	Mouse
*m*_*cyc*_	−	Hill coefficient for GC effect	∼ 2	Striped bass

The ratio *α*_*max*_/*α*_*min*_ = ∞ was effectively adopted in [37] by their choice of mathematical model, since cyclase activity vanishes as [Ca^2+^] → ∞ in that framework. The measurements for *α*_*max*_ were given for striped bass [37] and implicitly for carp in [30]. There the volumic concentrations of guanylyl cyclase and the Ca^2+^ sensing GCAPS were reported as [GC]_*vol*_ = 72 *μM* and [GCAP]_*vol*_ = 33 *μM*. The value *α*_*min*_ in carp cone was estimated as *α*_*min*_ = (72 *μM* GC)(1.7 cGMP formed/1GC/*s*) where the latter was the reported activity rate [30]. Similarly *α*_*max*_ was estimated assuming that all available GCAP was bound to GC and its reported activity rate:

$$\begin{array}{c} \alpha_{max}=\left(39\mu M\;\text{GC}\right)\frac{\left(1.7\;\text{cGMP}\;\text{formed}\right)}{\left(1\;\text{GC}\cdot s\right)}+\left(33\;\mu M\;\text{GC}:\text{GCAP}\right)\frac{\left(5.7\;\text{cGMP}\;\text{formed}\right)}{\left(1\;(\text{GC}:\text{GCAP})\cdot s\right)}. \end{array}$$

Together, these considerations estimated that the ratio *α*_*max*_/*α*_*min*_ ∼ 2. For mouse cones, GCAP1 normally dominates GCAP2 for binding of GC [40]. GCAP1 binding affinities were reported in [41].

#### Ionic current parameters

Table 5 reports the parameters for ionic current with experimental ranges given in the literature for striped bass primarily [33, 37, 42–44]. These inform the model through the equations

$$\begin{array}{c} D_{Ca^{2+}}\nabla [{\text{Ca}}^{2+}]\cdot n=\frac{1}{B_{Ca^{2+}}F}(\frac{f_{Ca^{2+}}}{2}\frac{J_{\text{cG}}^{max}}{\Sigma_{S}}\frac{{\left[\text{cG}\right]}^{m_{\text{cG}}}}{{\left[\text{cG}\right]}^{m_{\text{cG}}}+K_{\text{cG}}^{m_{\text{cG}}}}-\frac{J_{ex}^{sat}}{\Sigma_{S}}\frac{\left[{\text{Ca}}^{2+}\right]}{K_{ex}+[{\text{Ca}}^{2+}]}). \end{array}$$ (7)

**Table 5:** Parameters for ionic currents of cone outer segments.

	Unit	Description	Range	Species
$J_{\text{cG}}^{max}$	*pA*	CNG channel current at saturating [cG]	∼ 2500	Striped bass
*K*_*cG*_	*μM*	[cG] for half-maximal CNG channel opening	∼ 20	Mouse rod
*m*_*cG*_	−	Hill coefficient for CNG channel	∼ 2.5	Striped bass
$J_{ex}^{sat}$	*pA*	Exchanger current at saturating [Ca^2+^]	[3.0, 6.8]	Striped bass
*K*_*ex*_	*μM*	[Ca^2+^] for half-maximal exchanger activity	∼ 0.019	Striped bass
*f*_*Ca* ^2+^_	−	Fraction of current carried by Ca^2+^	[.25, .41]	Striped bass
*B*_*Ca* ^2+^_	−	Buffering power of cytoplasm for Ca^2+^	∼ 20	Mouse rod
*J*_*dark*_	*pA*	Dark current	[16.8, 37.8]	Striped bass
[cG]_*dark*_	*μM*	Concentration of cGMP in the dark	[2, 4]	Mouse rod
[Ca^2+^]_*dark*_	*μM*	Concentration of Ca^2+^ in the dark	0.4	Striped bass

**Fig 1:**
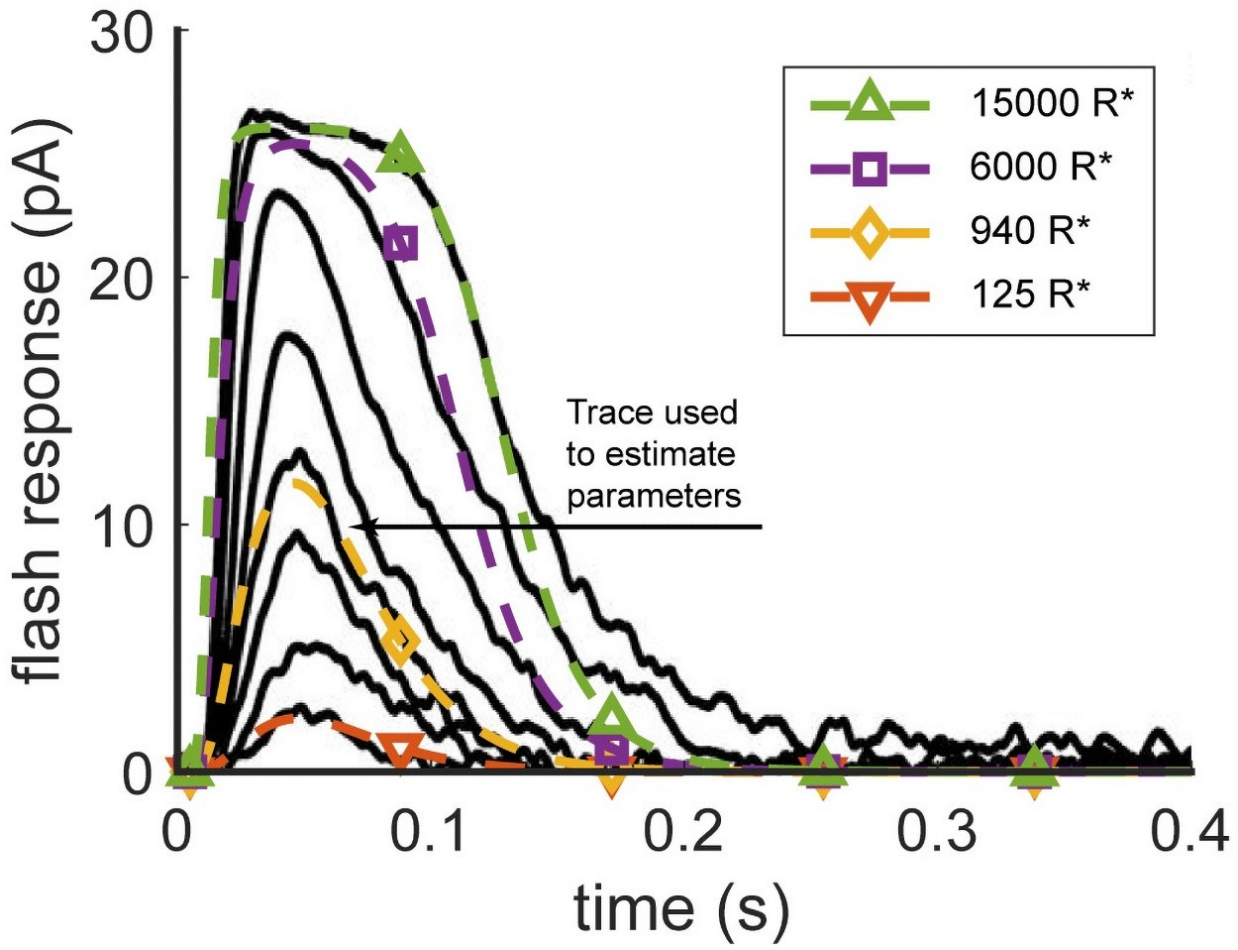
Modeling flash responses of a mouse cone. Black traces are flash responses recorded from a Gnat1^−/−^ mouse cone for an estimated range of 40—6,000 photoisomerizations with a half-maximal intensity that produced 940 photoisomerizations [45]. The colored traces show model predictions for indicated flash intensities. All simulations use the single set of parameters in Table 10. This set was found by stochastically minimizing the rms error between the model and experimental response solely for the 940-photoisomerizations trace while constraining the parameter values to satisfy known experimental constraints.

Here F stands for the Faraday constant and Σ_*S*_ is the surface area over which ion channels are distributed. Unless otherwise stated, channels were uniformly distributed over the sliver’s lateral boundary. The value *K*_*ex*_ = 19 *nM* was estimated numerically by [42] and is more than an order of magnitude smaller than the range 0.9 − 1.6 *μM* given for mouse rod. It is also evident from Fig 1 that, for the mouse cone examined in [45], *J*_*dark*_ ∼ 25.75 − 27 *pA* while circulating dark current for striped bass was reported as *J*_*dark*_ = 27.3 ± 10.5 *pA* [37, 42]. Finally, *K*_*cG*_ = 20 *μM* was taken from mouse rod [3]. For striped bass, *K*_*cG*_ was reported in [37, 43] to depend sigmoidally on Ca^2+^ and across the range 105 *μM* − 316 *μM*. Since this range was used in tandem with a high, computed dark cGMP concentration, [cG]_*dark*_ = 27.9 *μM*, we used the mouse rod value of [3] as other authors have reported [cG]_*dark*_ to be similar between rods and cones [30, 46]. The Ca^2+^-buffer, *B*_*Ca*^2+^_, is reported for mouse rod; however, this value is similar to that in [37] except there a functional relationship is used instead of a single value.

#### Diffusion coefficients

The diffusion coefficients for the G-protein machinery and second messengers (Table 6) were taken as those reported for mouse rod [3]. However, membrane diffusion is likely to be slower in cones than in rods because of the lower unsaturated fatty acid content in the cone outer segments [47]. Indeed, the diffusion coefficient for visual pigment was found to be approximately 16% lower in catfish cones than in amphibian red rods [48].

**Table 6:** Diffusion coefficients for cascade components in the membrane and for second messengers in the cytoplasm.

	Unit	Description	Range	Species
*D*_*R*_	*μm*^2^ *s*^−1^	Diffusion coefficient for R*	∼ 1.5	Mouse rod
*D*_*G*_	*μm*^2^ *s*^−1^	Diffusion coefficient for G*	∼ 2.2	Mouse rod
*D*_*E*_	*μm*^2^ *s*^−1^	Diffusion coefficient for PDE*	∼ 1.2	Mouse rod
*D*_*cG*_	*μm*^2^ *s*^−1^	Diffusion coefficient for cGMP	[50, 196]	Mouse rod
*D*_*Ca* ^2+^_	*μm*^2^ *s*^−1^	Diffusion coefficient for Ca^2+^	∼ 15	Mouse rod

#### Choice of ranges for Markov Chain Monte Carlo

The experimental ranges in Tables 3–6 are the basis for a Metropolis-Hastings Markov Chain Monte Carlo optimization scheme for estimating parameter values from experimental data. The MCMC stationary distribution penalized some parameter values if they did not belong to the ranges in Table 8 while other parameters were fully constrained to belong to the ranges in Table 7.

**Table 7:** Strict constraints imposed on many parameters that influence the dark current and the values derived from those parameters. The range for dark current was centered about the experimental flash response of [45], also shown in Fig 1.

Constraint Ranges	Units	Min	Max
[cG]_*dark*_	*μM*	2	4
[Ca^2+^]_*dark*_	*μM*	0.2	0.29
*J*_*dark*_	pA	25.75	27
[PDE]_*σ*_	*μm* ^−2^	0	750
*α*_*max*_	*μM s* ^−1^	50	500
*α*_*max*_/*α*_*min*_	-	2	20
*m*_*cyc*_	-	2	2.5
*K*_*cyc*_	nM	130	140
$J_{\text{cG}}^{max}$	pA	1000	5000
*m*_*cG*_	-	2	3.5
$J_{ex}^{sat}$	pA	1	10

**Table 8:** Parameter ranges within which there was no penalty imposed by the Metropolis-Hastings search. The geometric parameters were held fixed at *R*_*b*_ = 0.6 *μm*, *R*_*t*_ = 0.4 *μm*, *H* = 13.4 *μm*, *ϵ*_0_ = 16.8 *nm*, *ν* = 0.65, *ω*_0_ = *π*, and the Ca^2+^ diffusion coefficient was held constant at *D*_*Ca*^2+^_ = 15 *μm*^2^ *s*^−1^. [R]_*σ*_ was omitted since flash response depended only on the initial population of R*, and was otherwise independent of its surface density.

Expected Ranges	Units	Min	Max
**Catalytic activity and diffusion**			
*k*_*GE*_	*μm*^2^ *s*^−1^	0.05	1
*ν*_*RG*_	*s* ^−1^	30	330
*k*_*R*_	*s* ^−1^	1	200
*k*_*E*_	*s* ^−1^	5	150
*D*_*R*_	*μm*^2^ *s*^−1^	1	2
*D*_*G*_	*μm*^2^ *s*^−1^	1.2	3.2
*D*_*E*_	*μm*^2^ *s*^−1^	0.8	1.6
*D*_*cG*_	*μm*^2^ *s*^−1^	50	196
[G]_*σ*_	*μm* ^−2^	500	1500
**cGMP synthesis and hydrolysis**			
[PDE]_*σ*_	*μm* ^−2^	10	100
*β*_*dark*_	*s* ^−1^	1	1000
*B*_*cG*_	−	1	2
*k*_*cat*_/*K*_*m*_	*μM*^−1^ *s*^−1^	190	1810
*α*_*max*_	*μM s* ^−1^	50	500
*α*_*max*_/*α*_*min*_	−	2	20
*m*_*cyc*_	−	2	2.5
*K*_*cyc*_	*nM*	130	140
**CNG channel and Ca^2+^ exchanger**			
*B*_*Ca* ^2+^_	−	10	30
$J_{\text{cG}}^{max}$	*pA*	1000	5000
*m*_*cG*_	−	2.5	3.5
*K*_*cG*_	*μM*	10	30
*f*_*Ca* ^2+^_	−	0.2	0.35
$J_{ex}^{sat}$	*pA*	1	10
*K*_*ex*_	*μM*	0.02	5

### Choice of ranges for global sensitivity analysis

The parameter ranges used for Markov Chain Monte Carlo in Tables 7 and 8 were slightly revised, once the best fit was obtained, to reflect the optimized parameter values (Table 10). The Sobol, global sensitivity analysis was conducted using ranges in Table 9, mostly enlarged from Table 8.

**Table 9:** Ranges over which parameters were varied when conducting Sobol sensitivity analysis.

Ranges	Units	Min	Max
**Geometry**			
*R*_*b*_	*μm*	0.585	0.615
*R*_*t*_	*μm*	0.38	0.42
*H*	*μm*	12.7	14.1
*ω*_0_	−	2.51	3.77
*ϵ*_0_	*nm*	16.4	17.2
*ν*	−	0.61	0.71
*σ*	−	0.8	1.2
**Catalytic activity and diffusion**			
*k*_*GE*_	*μm*^2^ *s*^−1^	0.05	1
*ν*_*RG*_	*s* ^−1^	30	330
*k*_*R*_	*s* ^−1^	1	200
*k*_*E*_	*s* ^−1^	5	150
*D*_*R*_	*μm*^2^ *s*^−1^	1	2
*D*_*G*_	*μm*^2^ *s*^−1^	1.1	3.2
*D*_*E*_	*μm*^2^ *s*^−1^	0.8	1.6
*D*_*cG*_	*μm*^2^ *s*^−1^	50	196
*D*_*Ca* ^2+^_	*μm*^2^ *s*^−1^	12	18
[G]_*σ*_	*μm* ^−2^	200	1500
**cGMP synthesis and hydrolysis**			
[PDE]_*σ*_	*μm* ^−2^	10	120
*β*_*dark*_	*s* ^−1^	1	1000
*B*_*cG*_	−	1	2
*k*_*cat*_/*K*_*m*_	*μM*^−1^ *s*^−1^	190	1810
*α*_*max*_	*μM s* ^−1^	50	500
*a*_*min*_	−	0	1
*m*_*cyc*_	−	2	2.5
*K*_*cyc*_	*nM*	130	140
**CNG channel and Ca^2+^ exchanger**			
*B*_*Ca* ^2+^_	−	10	30
$J_{\text{cG}}^{max}$	*pA*	1000	5000
*m*_*cG*_	−	2	3.5
*K*_*cG*_	*μM*	10	30
*f*_*Ca* ^2+^_	−	0.2	0.35
$J_{ex}^{sat}$	*pA*	1	10
*K*_*ex*_	*μM*	0.02	5

**Table 10:** Parameter values for a mouse cone found by minimizing the rms error between experiment and model predictions for a flash producing 940 photoisomerizations according to the Metropolis-Hastings random walk.

Symbol	Units	Definition	Value
*α*_*max*_	*μM s* ^−1^	Maximal rate of cGMP synthesis at low [Ca^2+^]	**55.8**
*α*_*max*_/*α*_*min*_	−	GC synthesis at low relative to high [Ca^2+^]	**5.51**
*β*_*dark*_	*s* ^−1^	Rate of cGMP hydrolysis by dark-activated PDE	**8.57**
*B*_*cG*_	−	Buffering power of cytoplasm for cGMP	**1.8**
*B*_*Ca* ^2+^_	−	Buffering power of cytoplasm for Ca^2+^	**25.3**
*k*_*GE*_	*μm*^2^ *s*^−1^	Coupling coefficient for PDE* formation by G*	**0.33**
[cG]_*dark*_	*μM*	Concentration of cGMP in the dark	**2.65**
[Ca^2+^]_dark_	*μM*	Concentration of Ca^2+^ in the dark	**0.204**
*R*_*b*_	*μm*	Radius of COS base	0.6
*R*_*t*_	*μm*	Radius of COS tip	0.4
*ω*_0_	−	Open margin angle for sliver	*π*
*D*_*cG*_	*μm*^2^ *s*^−1^	Diffusion coefficient for cGMP	**89.2**
*D*_*Ca* ^2+^_	*μm*^2^ *s*^−1^	Diffusion coefficient for Ca^2+^	15
*D*_*E*_	*μm*^2^ *s*^−1^	Diffusion coefficient for PDE*	**1.47**
*D*_*G*_	*μm*^2^ *s*^−1^	Diffusion coefficient for G*	**1.42**
*D*_*R*_	*μm*^2^ *s*^−1^	Diffusion coefficient for R*	**1.71**
*ϵ*_0_	*nm*	Disc thickness	16.8
*η*	*nm*	Volume to surface ratio	0.01
F	*C*/mol	Faraday’s constant	96500
*f*_*Ca* ^2+^_	−	Fraction of current carried by Ca^2+^	**0.26**
*H*	*μm*	Length of COS	13.4
*J*_*dark*_	*pA*	Dark current	**26.1**
$J_{\text{cG}}^{max}$	*pA*	CNG channel current at saturating [GC]	**3138**
$J_{ex}^{sat}$	*pA*	Exchanger current at saturating [Ca^2+^]	**8.79**
*k*_*cat*_/*K*_*m*_	*μM*^−1^ *s*^−1^	Hydrolytic efficiency of PDE* dimer	**540**
*k*_*σ*;*hyd*_	*μm*^3^ *s*^−1^	Surface hydrolysis rate of cGMP by dark-activated PDE	**4.1e-4**
$k_{\sigma ;hyd}^{*}$	*μm*^3^ *s*^−1^	Surface hydrolysis rate of cGMP by PDE*	**0.5**
*k*_*R*_	*s* ^−1^	Rate constant for inactivation of R*	**87.1**
*k*_*E*_	*s* ^−1^	Rate constant for inactivation of PDE*	**82.3**
*K*_*cyc*_	*nM*	Half-saturating [Ca^2+^] for GC activity	**134**
*K*_*cG*_	*μM*	[cG] for half-maximal CNG channel opening	**14.2**
*K*_*ex*_	*μM*	[Ca^2+^] for half-maximal exchanger activity	**0.4**
*ν*	−	Ratio between interdiscal space and disc thickness	0.65
$\nu_{\epsilon_{0}}$	*nm*	Interdiscal space thickness	11
*ν*_*RG*_	*s* ^−1^	Rate of G* formation per R*	**212**
*ν*_*GE*_	*s* ^−1^	Rate of PDE* formation per G*	**37.95**
*n*	−	Number of discs	400
*N*_Av_	*mol* ^−1^	Avogadro number	6.02*e*23
*m*_*cyc*_	−	Hill coefficient for GC effect	**2.3**
*m*_*cG*_	−	Hill coefficient for CNG channel	**2.9**
[G]_*σ*_	*μm* ^−2^	Surface density of G	**253.82**
[PDE]_*σ*_	*μm* ^−2^	Surface density of PDE	**115**
*σ*	−	Ratio between the disc thickness and sliver thickness	1
$\sigma_{\epsilon_{0}}$	*nm*	Distance between the disc rim and outer plasma membrane at sliver	16.8

Parameter values obtained by minimizing the rms error are shown in bold.

### Numerical implementation by finite elements

We used the homogenized, finite element model of cone phototransduction published in [16] with adjustments to handle the case of continuously distributed illumination such as for cone’s native bright light settings. The software library used in this work is available on GitHub [49], and the simulation data produced by it are available through Dryad repository [50]. See also S1 Appendix.

### Identifying a consistent set of parameters

To choose a consistent set of parameters in murine cones, the root-mean-square (rms) error between the experimental flash response reported in [45] and model prediction was minimized for 940 photoisomerizations (Fig 1). This flash intensity was chosen because it was the value reported by [45] for attaining a half-maximal response. Some parameter values were constrained to a subset, *K*, of parameter space defined by the ranges in Table 7. Since intervals for many biochemical parameters were unknown, exploration of parameter space for other parameter values outside of anticipated ranges was only penalized rather than prohibited (Table 8). Let *e*(*x*) stand for the error between the experimental flash response and the model prediction for a choice of parameter values *x*. A Metropolis-Hastings random-walk was used to construct a Markov chain with stationary probability distribution over parameter space with density *π*(*x*) satisfying the proportionality relation

$$\begin{array}{c} \pi \left(x\right)∼(\prod_{i}2^{-\gamma \text{dist}(x_{i},I_{i})}){\left\|e(x)\right\|}_{2}^{-\beta }1_{K}\left(x\right) \end{array}$$ (8)

for user-selected values *γ*, *β* > 0. (See also S1 Appendix). The interval *I*_*i*_ was the range reported in Table 8 for the i^th^ parameter. Sampling the Markov chain then preferentially explored regions of parameter space with high probability [22, 23]. By construction this was where the error, *e*(*x*), was small.

### Choice of functionals for sensitivity analysis

Sensitivity analysis was performed by measuring the change in certain quantities of the flash response, hereafter called functionals, that were elicited by changes in the parameter values. The following functionals were used to measure various aspects of the photoresponse: *I*_*act*_ was defined by first identifying the current drop for times *t* in the interval [0, 10 *ms*] with the quadratic function $\frac{1}{2}At^{2}$ and then setting *I*_*act*_ = *A*. *E*_*act*_ was similar but for the total amount of E* across the outer segment at any instant of time, where E* = 2[PDE*] is the concentration of active catalytic subunits of PDE. Each PDE had two such subunits, which in the model could be activated independently. *I*_*drop*_ was defined as the maximum drop in flash response attained at any instant of time expressed in proportion to the dark current. *E*_*peak*_ was the total amount of E* aggregated across the outer segment at any instant of time. A functional for the recovery phase of E* was also included. *E*_*rec*_ was found by fitting the current drop over the time interval [0.135 *s*, 0.5 *s*] with an exponential *ce*^−*αt*^ and then taking *E*_*rec*_ = *α*. The analog for current drop was not included because an exponential does not correctly model cone overshoot. *J*_*dark*_ and *T*_*peak*_ were more simply the circulating dark current and time-to-peak of the current drop. *J*_*over*_ was the greatest magnitude of overshoot (nonpositive) current values exhibited by the flash response expressed in proportion to the dark current. In addition to these we also introduced an error functional, denoted *L*^2^. This functional quantified the rms error between the experimental flash response reported by [45] and the model prediction for the flash producing 940 isomerizations.

### Local sensitivity analysis

Once a choice of parameter values was made, $x^{*}=(x_{1}^{*},\ldots ,x_{m}^{*})$, the local sensitivity of the model for a functional *y* at x* was computed with a gradient-based method [3] by the quantity

$$\begin{array}{c} Q_{i}=\frac{{\left(\partial y/\partial x_{i}\right)}_{|x=x^{*}}}{y_{|x=x^{*}}/x_{i}}. \end{array}$$

*Q*_*i*_ measures the instantaneous relative change in the functional *y* with respect to the relative change in parameter *x*_*i*_ based at the point x*.

### Global sensitivity analysis

In contrast to local sensitivity analysis, which is based on a specific choice of parameter values, global sensitivity analysis examines the statistical variance of a functional, when its input parameters vary *independently*, in the statistical sense, over given ranges. Using the GSA method of Sobol indices [17, 18, 51], a functional was decomposed into components whose own respective variances quantified interactions between subgroups of parameters. This analysis precisely determined the percentage of the functional’s total statistical variance which was due to interactions between the freely chosen, prefixed subset of parameters. In this work, we considered the indices *S*_*y*_ and $S_{y}^{tot}$ where *y* was either a single parameter *x*_*i*_ or was a collection of two parameters *x*_*i*_, *x*_*j*_. The index *S*_*y*_ was the percentage of total variance that could be explained by the parameters in *y* alone while $1-S_{y}^{tot}$ was the percentage of total variance that could be explained using only parameters not in *y*. Equivalently, $S_{y}^{tot}$ was the fraction of variance that was due to the parameters in *y* interacting with all other parameters when they were randomly varied at once. It follows that $S_{y}\leq S_{y}^{tot}$. It further holds that as *S*_*y*_ approaches 1, the functional tends to only depend on the parameters in *y*. Similarly as $S_{y}^{tot}$ approaches 0, the functional tends to not depend on the parameters in *y*. Owing to the inherent randomness in parameters that define biological systems, GSA is an apt tool for quantifying the significance of parameters upon model output. (See also S1 Appendix).

The Sobol indices were estimated by Monte Carlo integration through a scheme first presented in [25]. See also [26]. In total, 6.8 million Monte Carlo trials were performed at the Ohio Supercomputer Center [52], so that each statistic needed for Sobol analysis was estimated using 100, 000 trials. Confidence intervals were constructed assuming that sufficiently many trials had been conducted so that the corresponding sample means were normally distributed. The validity of this assumption was investigated using a bootstrap sampling procedure (*e.g*. [53]) of the empirical samples. This procedure along with convergence rates and confidence intervals are given in S1 Appendix. Since the Sobol indices were ratios of two Monte Carlo estimated quantities, after the numerators and denominators were given 95% confidence intervals, the indices were then estimated up to 90% confidence using also the order-preserving properties of division.

#### Renormalizing *α*_*min*_ to *a*_*min*_

The Sobol method required that the considered parameters be *independently* sampled. However, the existence of a dark-adapted steady state required a first-principles balance of fluxes between the CNG channel and exchanger currents as well as a balance between cGMP synthesis and hydrolysis in the dark:

$$\begin{array}{cc} \beta_{dark}{\left[\text{cG}\right]}_{dark} & =(\alpha_{min}+\left(\alpha_{max}-\alpha_{min}\right)\frac{K_{cyc}^{m_{\text{cyc}}}}{K_{cyc}^{m_{\text{cyc}}}+{{\left[{\text{Ca}}^{2+}\right]}_{dark}}^{m_{\text{cyc}}}}) \end{array}$$ (9)

$$\begin{array}{cc} J_{ex}^{sat}\frac{{\left[{\text{Ca}}^{2+}\right]}_{dark}}{K_{ex}+{\left[{\text{Ca}}^{2+}\right]}_{dark}} & =J_{\text{cG}}^{max}\frac{f_{Ca^{2+}}}{2}\frac{{{\left[\text{cG}\right]}_{dark}}^{m_{\text{cG}}}}{K_{cG}^{m_{\text{cG}}}+{{\left[\text{cG}\right]}_{dark}}^{m_{\text{cG}}}}. \end{array}$$ (10)

In particular, Eqs 9 and 10 could not be satisfied for arbitrary parameter choices. A criterion for the existence of the two dark concentrations [Ca^2+^]_*dark*_ and [cG]_*dark*_ was the constraint [3]

$$\begin{array}{c} 1+{\left(\frac{\alpha_{min}}{\beta_{dark}K_{cG}}\right)}^{m_{cG}}<{\left(1-\frac{2J_{ex}^{sat}}{J_{\text{cG}}^{max}f_{Ca^{2+}}}\right)}^{-1}. \end{array}$$ (11)

This constraint constituted a dependence between the constituent parameters, and a change of variables was required to reestablish a parameter set compatible with independent sampling. Once ranges for all parameters except *α*_*min*_ were fixed, the monotonicity of Eq 11 ensured that a steady state would exist so long as *α*_*min*_ belonged to the interval [0, *ξ*]. Here $\xi =\text{min}\{\alpha_{max},\chi \left(\beta_{dark},K_{cG},m_{cG},J_{ex}^{sat},J_{\text{cG}}^{max},f_{Ca^{2+}}\right)\}$ where *χ* satisfies the equality

$$\begin{array}{c} 1+{\left(\frac{\chi }{\beta_{dark}K_{cG}}\right)}^{m_{cG}}={\left(1-\frac{2J_{ex}^{sat}}{J_{\text{cG}}^{max}f_{Ca^{2+}}}\right)}^{-1}. \end{array}$$ (12)

We defined the unitless parameter

$$\begin{array}{c} a_{min}≔\frac{\alpha_{min}}{\xi (\alpha_{max},\beta_{dark},K_{cG},m_{cG},J_{ex}^{sat},J_{\text{cG}}^{max},f_{Ca^{2+}})}. \end{array}$$ (13)

No further information on the biological uncertainty of this parameter was assumed, and so it was taken to be uniformly distributed over the interval [0, 1]. The value of *a*_*min*_ represents the *relative position* of *α*_*min*_ within the interval [0, *ξ*] over which the dark steady state exists. In particular, as *a*_*min*_ approaches either 0 or 1, *α*_*min*_ approaches extremes where the existence of a steady state becomes impossible. Through this change of variables, the new collection of parameters with *α*_*min*_ replaced by *a*_*min*_ could be sampled independently for Sobol analysis and the model evaluated through the substitution $\alpha_{min}=\xi \left(\alpha_{max},\beta_{dark},K_{cG},m_{cG},J_{ex}^{sat},J_{\text{cG}}^{max},f_{Ca^{2+}}\right)a_{min}$.

## Results

### Consistent parameter set

The Markov chain was sampled to obtain the best fit with values shown in Table 10 for a Gnat1^−/−^ cone (Fig 1). Knockout of rod transducin in the Gnat1^−/−^ mouse rendered rods nonfunctional [54], thereby precluding any intrusion of rod responses in the mouse cone recordings (*e.g*., [55]).

This fit was performed for a response to a flash producing 940 photoisomerizations, which was reported by [45] to be at half-maximal flash response. Parameter values were subject to the constraints in Table 7 and penalized if they fell outside the ranges in Table 8. Because the distribution, Eq 8, was chosen to prioritize exploration of quality fitting regions of parameter space, and was not, for example, used in a Bayesian framework [56, 57], the chain was not necessarily sampled until a stationary distribution was attained. For convenience, the deposited software library may be used to continue the MCMC chain when desired. Modeling results for striped bass cone flash responses subject to the same biochemical ranges of Table 9 are included in S1 Appendix as supplementary materials.

### Local sensitivity of parameters

Table 11 reports the local sensitivity of functionals *y*(*x*) at *x* = x* with respect to the coordinate *x*_*i*_ as described in **Methods**. The partial derivatives were numerically computed by increasing the *x*_*i*_ parameter by a relative 5% when forming the numerical difference quotient.

**Table 11:** Local sensitivity indices for a flash of 940 isomerizations uniformly distributed throughout the outer segment.

Local Sensitivity	*E*_*act*_	*E*_*peak*_	*E*_*act*_	*I*_*act*_	*I*_*drop*_	*T*_*peak*_	*J*_*dark*_	*L* ^2^
**Geometry**								
*R*_*b*_	0.00	0.00	0.00	-1.14	-0.75	0.00	0.00	0.01
*R*_*t*_	0.00	0.00	0.00	-0.68	-0.45	0.00	0.00	0.00
*H*	0.00	0.00	0.00	-0.94	-0.66	0.00	0.00	0.01
*ω*_0_	0.00	0.00	0.00	0.00	0.00	0.00	0.00	0.00
*ϵ*_0_	0.00	0.00	0.00	-0.12	-0.03	0.00	0.00	0.00
*ν*	0.00	0.00	0.00	0.06	0.02	0.00	0.00	0.00
*σ*	0.00	0.00	0.00	-0.12	-0.03	0.00	0.00	0.00
**Catalytic activity and diffusion**								
*k*_*GE*_	0.78	0.34	0.58	0.82	0.16	-0.18	0.00	0.00
*ν*_*RG*_	1.00	1.00	0.00	0.97	0.69	0.00	0.00	0.01
*k*_*R*_	-0.23	-0.66	0.12	-0.17	-0.54	-0.18	0.00	0.01
*k*_*E*_	-0.22	-0.65	0.21	-0.16	-0.53	-0.18	0.00	0.01
*D*_*R*_	0.00	0.00	0.00	0.00	0.00	0.00	0.00	0.00
*D*_*G*_	0.00	0.00	0.00	0.00	0.00	0.00	0.00	0.00
*D*_*E*_	0.00	0.00	0.00	0.00	0.00	0.00	0.00	0.00
*D*_*cG*_	0.00	0.00	0.00	0.02	0.00	0.00	0.00	0.00
*D*_*Ca* ^2+^_	0.00	0.00	0.00	-0.01	-0.05	0.00	0.00	0.00
[G]_*σ*_	0.00	0.00	0.00	0.00	0.00	0.00	0.00	0.00
**cGMP synthesis and hydrolysis**								
[PDE]_*σ*_	0.78	0.35	0.58	0.82	0.16	-0.18	0.00	0.00
*β*_*dark*_	0.00	0.00	0.00	-0.58	-0.14	0.00	-0.56	0.01
*B*_*cG*_	0.00	0.00	0.00	-0.93	-0.67	0.00	0.00	0.01
*k*_*cat*_/*K*_*m*_	0.00	0.00	0.00	0.97	0.69	0.00	0.00	0.01
*α*_*max*_	0.00	0.00	0.00	0.35	-0.33	-0.36	0.38	0.00
*α*_*min*_	0.00	0.00	0.00	0.20	0.10	0.00	0.19	0.00
*m*_*cyc*_	0.00	0.00	0.00	-0.24	-0.30	-0.18	-0.21	0.01
*K*_*cyc*_	0.00	0.00	0.00	0.51	-0.01	0.00	0.52	0.00
**CNG channel and Ca^2+^ exchanger**								
*B*_*Ca* ^2+^_	0.00	0.00	0.00	-0.01	0.01	0.00	0.00	0.00
$J_{\text{cG}}^{max}$	0.00	0.00	0.00	0.20	-0.08	0.00	0.19	0.00
*m*_*cG*_	0.00	0.00	0.00	0.02	0.89	0.18	-0.91	0.00
*K*_*cG*_	0.00	0.00	0.00	-0.54	0.26	0.18	-0.56	0.00
*f*_*Ca* ^2+^_	0.00	0.00	0.00	-0.67	-0.08	0.00	-0.66	0.01
$J_{ex}^{sat}$	0.00	0.00	0.00	0.81	0.07	0.00	0.80	0.01
*K*_*ex*_	0.00	0.00	0.00	-0.51	0.03	0.00	-0.51	0.01

### Global sensitivity of parameters

Owing to the number of model parameters and the uncertainty in their experimental values, global sensitivity analysis was performed to assess which uncertainties had the biggest effect upon model output. Parameter values were uniformly sampled across the ranges given in Table 9. For each choice of parameters, the model simulated the response to the flash of 940 isomerizations. Associated functionals that quantified individual components of the cascade were computed along with their statistical variance. The Sobol method then assigned percentages of that variance that were due to individual and subcollections of parameters. This allowed the parameters to be ranked by their effect across all ranges of uncertainty (Table 9). Hereafter, these percentages are reported as numbers in the interval [0, 1].

#### Findings

**Fig 2:**
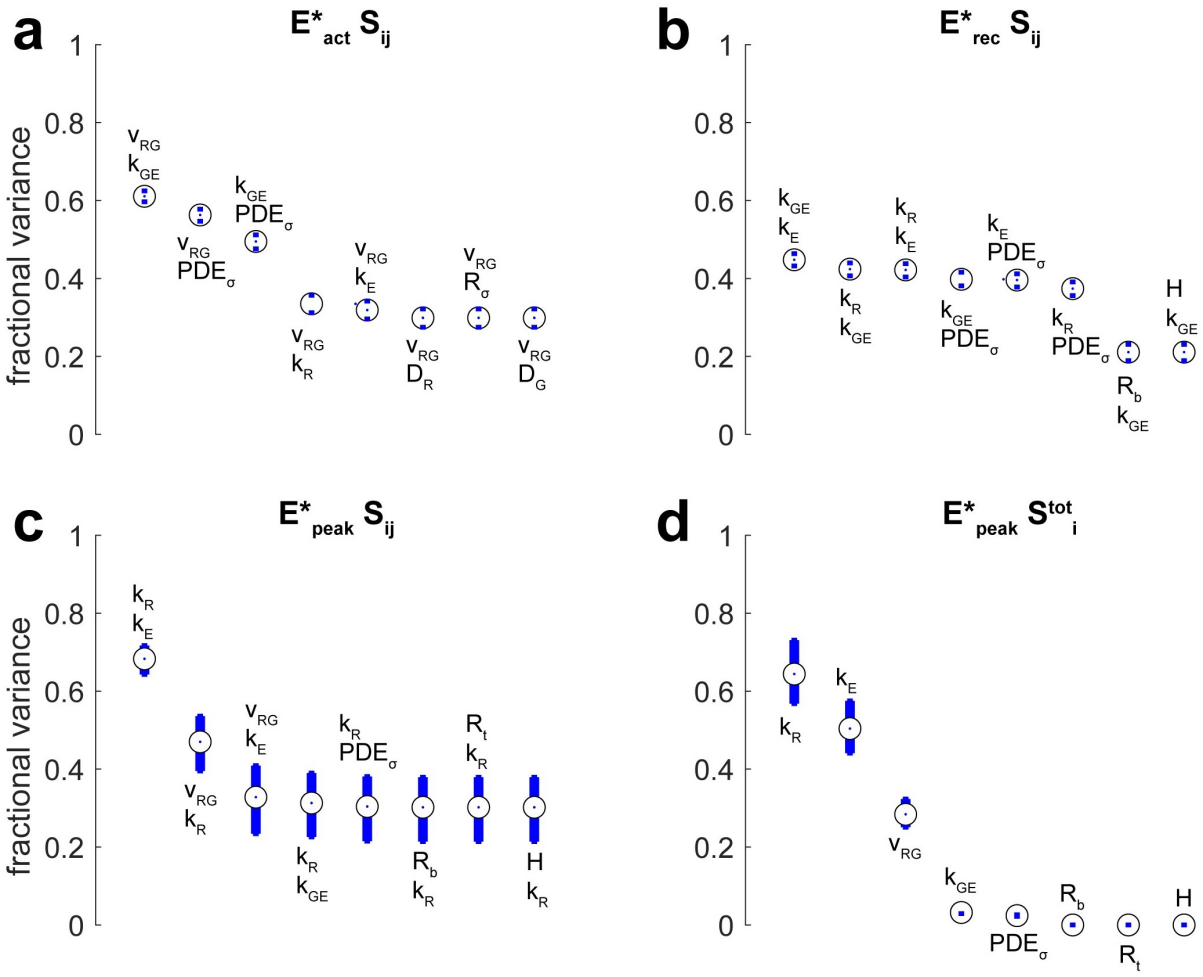
Sobol indices for functionals quantifying E*. The dot at the center of a circle is the Sobol index obtained by Monte Carlo evaluation (100,00 samples). The blue bars define a 90% confidence interval. Plots show the eight most influential parameters ordered from most significant to least significant. **(a)** Pairwise sensitivity indices for E* activation. **(b)** Pairwise sensitivity indices for E* recovery. **(c)** Pairwise sensitivity indices for peak E* production. **(d)** Total sensitivity indices for peak E* production.

**Fig 3:**
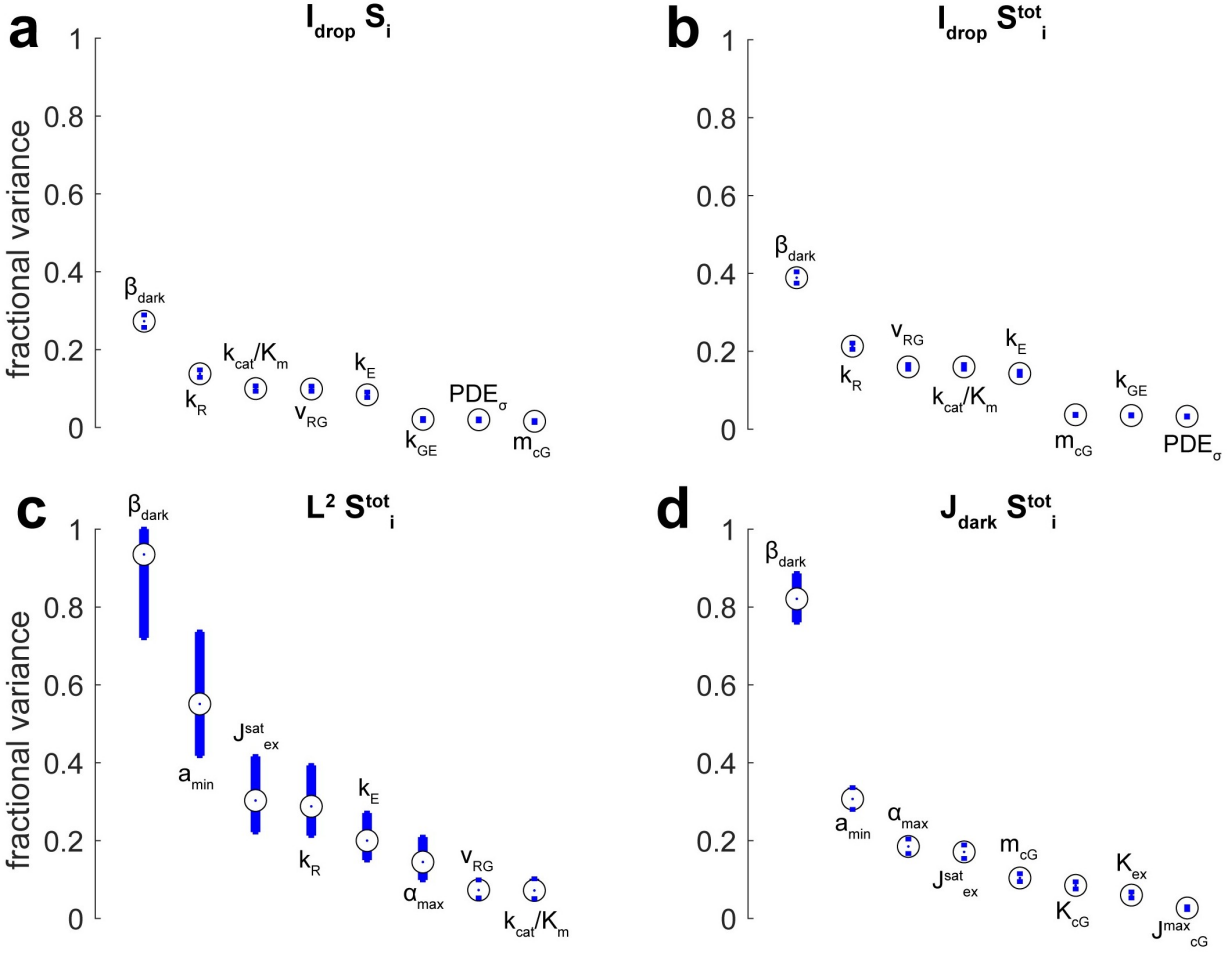
Sobol indices for functionals quantifying the drop in current due to flash response, the rms error between simulation and experiment, and the dark current. Blue bars define a 90% confidence interval (100,000 samples). Plots show the eight most influential parameters. Confidence intervals could be off-center of the estimated Sobol index, because the indices were ratios of two Monte Carlo estimated quantities. **(a)** Single sensitivity indices for the current drop. **(b)** Total sensitivity indices for the current drop. **(c)** Total sensitivity indices for the rms error between model prediction and experiment. **(d)** Total sensitivity indices for the circulating dark current.

**Fig 4:**
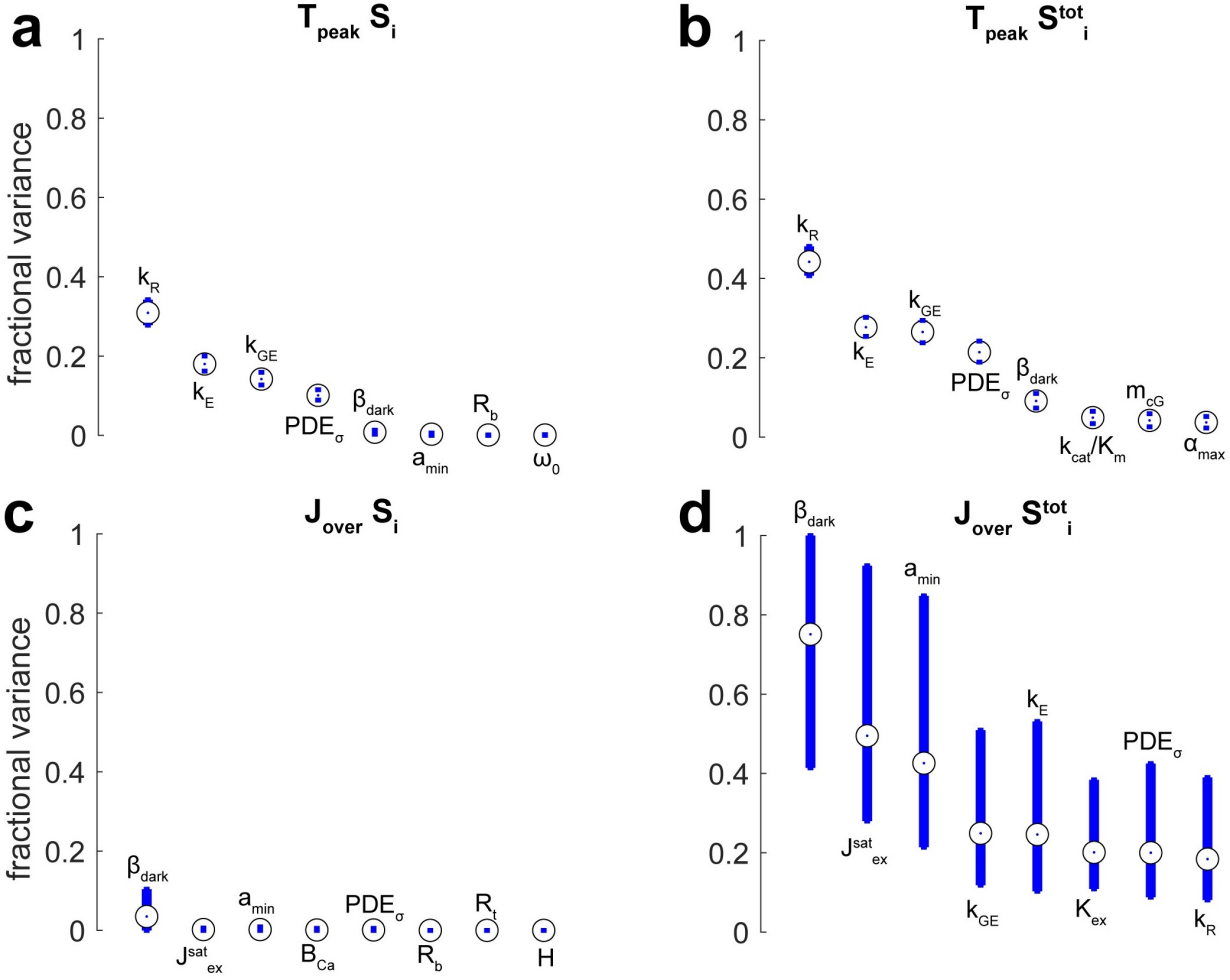
Sobol indices for functionals quantifying the time-to-peak of the current drop and its overshoot. Blue bars define a 90% confidence interval (100,000 samples). Plots show the eight most influential parameters. Confidence intervals could be off-center of the estimated Sobol index, because the indices were ratios of two Monte Carlo estimated quantities. **(a)** Single sensitivity indices for the time-to-peak. **(b)** Total sensitivity indices for the time-to-peak. **(c)** Single sensitivity indices for the overshoot. **(d)** Total sensitivity indices for the overshoot.

Tables 12 and 13 and Figs 2–4 report global sensitivity of functionals using the Sobol method described in **Methods** (100,000 samples per estimate) for ranges of parameters in Table 9. The eight most influential (pairs of) parameters are shown for the given functional. The Monte Carlo Sobol trials along with additional convergence rates and confidence intervals are available at Dryad [50].

**Table 12:** Single sensitivity indices. As an index approached 1, the functional became dependent only on that parameter. Most values shown are close to 0, which indicated that nonlinear interactions between parameters dominated the cone flash response. While the theoretical value of the Sobol index must fall in the interval [0, 1], small negative values sometimes occurred above as an artifact of the Monte Carlo approximation. These should be regarded as approximately 0. Confidence intervals are given in S1 Appendix.

*S*_*i*_	*E*_*act*_	*E*_*peak*_	*E*_*rec*_	*I*_*act*_	*I*_*drop*_	*T*_*peak*_	*J*_*dark*_	*J*_*over*_	*L* ^2^
**Geometry**									
*R*_*b*_	0.00	0.00	0.00	0.00	0.00	0.00	0.00	0.00	0.00
*R*_*t*_	0.00	0.00	0.00	0.00	0.00	0.00	0.00	0.00	0.00
*H*	0.00	0.00	0.00	0.00	0.00	0.00	0.00	0.00	0.00
*ω*_0_	0.00	0.00	0.00	0.00	0.00	0.00	0.00	0.00	0.00
*ϵ*_0_	0.00	0.00	0.00	0.00	0.00	0.00	0.00	0.00	0.00
*ν*	0.00	0.00	0.00	0.00	0.00	0.00	0.00	0.00	0.00
*σ*	0.00	0.00	0.00	0.00	0.00	0.00	0.00	0.00	0.00
**Catalytic activity and diffusion**									
*k*_*GE*_	0.25	0.02	0.21	0.01	0.02	0.14	0.00	0.00	0.00
*ν*_*RG*_	0.30	0.11	0.00	0.01	0.10	0.00	0.00	0.00	-0.01
*k*_*R*_	0.03	0.28	0.19	0.00	0.14	0.31	0.00	0.00	0.00
*k*_*E*_	0.02	0.16	0.20	0.00	0.08	0.18	0.00	0.00	0.00
*D*_*R*_	0.00	0.00	0.00	0.00	0.00	0.00	0.00	0.00	0.00
*D*_*G*_	0.00	0.00	0.00	0.00	0.00	0.00	0.00	0.00	0.00
*D*_*E*_	0.00	0.00	0.00	0.00	0.00	0.00	0.00	0.00	0.00
*D*_*cG*_	0.00	0.00	0.00	0.00	0.00	0.00	0.00	0.00	0.00
*D*_*Ca* ^2+^_	0.00	0.00	0.00	0.00	0.00	0.00	0.00	0.00	0.00
[G]_*σ*_	0.00	0.00	0.00	0.00	0.00	0.00	0.00	0.00	0.00
**cGMP synthesis and hydrolysis**									
[PDE]_*σ*_	0.22	0.01	0.16	0.01	0.02	0.10	0.00	0.00	0.00
*β*_*dark*_	0.00	0.00	0.00	0.20	0.27	0.01	0.37	0.04	0.06
*B*_*cG*_	0.00	0.00	0.00	0.00	0.01	0.00	0.00	0.00	0.00
*k*_cat_/*K*_*m*_	0.00	0.00	0.00	0.01	0.10	0.00	0.00	0.00	0.00
*α*_*max*_	0.00	0.00	0.00	0.01	0.00	0.00	0.03	0.00	0.01
*a*_*min*_	0.00	0.00	0.00	0.01	0.00	0.00	0.03	0.00	-0.02
*m*_*cyc*_	0.00	0.00	0.00	0.00	0.00	0.00	0.00	0.00	0.00
*K*_*cyc*_	0.00	0.00	0.00	0.00	0.00	0.00	0.00	0.00	0.00
**CNG channel and Ca^2+^ exchanger**									
*B*_*Ca* ^2+^_	0.00	0.00	0.00	0.00	0.00	0.00	0.00	0.00	0.00
$J_{\text{cG}}^{max}$	0.00	0.00	0.00	0.00	0.00	0.00	0.00	0.00	0.00
*m*_*cG*_	0.00	0.00	0.00	0.00	0.02	0.00	0.02	0.00	0.00
*K*_*cG*_	0.00	0.00	0.00	0.00	0.00	0.00	0.01	0.00	-0.01
*f*_*Ca* ^2+^_	0.00	0.00	0.00	0.00	0.00	0.00	0.00	0.00	-0.01
$J_{ex}^{sat}$	0.00	0.00	0.00	0.01	0.00	0.00	0.01	0.00	0.00
*K*_*ex*_	0.00	0.00	0.00	0.00	0.00	0.00	0.01	0.00	0.00

**Table 13:** Total sensitivity indices. As an index approached 0, the functional became essentially independent of that parameter. A large index value indicated that the considered parameter contributed to significant nonlinear interactions with other model parameters, so that ignoring it would amount to that index’s loss, as a proportion, of the total variance. Some parameters that were negligible, *e.g*. *m*_*cyc*_, may have been so because their prescribed uncertainties were smaller than other parameters. Confidence intervals are given in S1 Appendix.

$S_{i}^{tot}$	*E*_*act*_	*E*_*peak*_	*E*_*rec*_	*I*_*act*_	*I*_*drop*_	*T*_*peak*_	*J*_*dark*_	*J*_*over*_	*L* ^2^
**Geometry**									
*R*_*b*_	0.00	0.00	0.00	0.00	0.00	0.00	0.00	0.00	0.00
*R*_*t*_	0.00	0.00	0.00	0.00	0.00	0.01	0.00	0.00	0.00
*H*	0.00	0.00	0.00	0.00	0.00	0.00	0.00	0.00	0.00
*ω*_0_	0.00	0.00	0.00	0.00	0.00	0.01	0.00	0.05	0.00
*ϵ*_0_	0.00	0.00	0.00	0.00	0.00	0.01	0.00	0.00	0.00
*ν*	0.00	0.00	0.00	0.00	0.00	0.01	0.00	0.01	0.00
*σ*	0.00	0.00	0.00	0.00	0.00	0.01	0.00	0.00	0.00
**Catalytic activity and diffusion**									
*k*_*GE*_	0.37	0.03	0.35	0.17	0.04	0.27	0.00	0.25	0.01
*ν*_*RG*_	0.43	0.28	0.00	0.17	0.16	0.01	0.00	0.09	0.07
*k*_*R*_	0.05	0.64	0.32	0.01	0.21	0.44	0.00	0.18	0.29
*k*_*E*_	0.03	0.50	0.35	0.01	0.14	0.28	0.00	0.25	0.20
*D*_*R*_	0.00	0.00	0.00	0.00	0.00	0.00	0.00	0.00	0.00
*D*_*G*_	0.00	0.00	0.01	0.00	0.00	0.00	0.00	0.00	0.00
*D*_*E*_	0.00	0.00	0.00	0.00	0.00	0.00	0.00	0.00	0.00
*D*_*cG*_	0.00	0.00	0.00	0.00	0.00	0.02	0.00	0.00	0.00
*D*_*Ca* ^2+^_	0.00	0.00	0.00	0.00	0.00	0.00	0.00	0.00	0.00
[G]_*σ*_	0.00	0.00	0.00	0.00	0.00	0.00	0.00	0.00	0.00
**cGMP synthesis and hydrolysis**									
[PDE]_*σ*_	0.32	0.02	0.29	0.15	0.03	0.21	0.00	0.20	0.00
*β*_*dark*_	0.00	0.00	0.00	0.86	0.39	0.09	0.82	0.75	0.94
*B*_*cG*_	0.00	0.00	0.00	0.03	0.02	0.03	0.00	0.02	0.01
*k*_cat_/*K*_*m*_	0.00	0.00	0.00	0.18	0.16	0.05	0.00	0.16	0.07
*α*_*max*_	0.00	0.00	0.00	0.16	0.02	0.04	0.19	0.03	0.15
*a*_*min*_	0.00	0.00	0.00	0.29	0.03	0.03	0.31	0.43	0.55
*m*_*cyc*_	0.00	0.00	0.00	0.00	0.00	0.03	0.00	0.01	0.00
*K*_*cyc*_	0.00	0.00	0.00	0.00	0.00	0.03	0.00	0.00	0.00
**CNG channel and Ca^2+^ exchanger**									
*B*_*Ca* ^2+^_	0.00	0.00	0.00	0.00	0.00	0.01	0.00	0.13	0.00
$J_{\text{cG}}^{max}$	0.00	0.00	0.00	0.02	0.00	0.03	0.03	0.01	0.02
*m*_*cG*_	0.00	0.00	0.00	0.05	0.04	0.04	0.10	0.04	0.05
*K*_*cG*_	0.00	0.00	0.00	0.06	0.01	0.03	0.09	0.02	0.05
*f*_*Ca* ^2+^_	0.00	0.00	0.00	0.02	0.00	0.03	0.02	0.00	0.04
$J_{ex}^{sat}$	0.00	0.00	0.00	0.19	0.00	0.03	0.17	0.50	0.30
*K*_*ex*_	0.00	0.00	0.00	0.06	0.01	0.03	0.06	0.20	0.07

Sensitivity indices for E* production are given in Fig 2. Monte Carlo estimated that *k*_*R*_ and *k*_*E*_ alone accounted for approximately 70% of the variance in peak E* production while *ν*_*RG*_ also played an influential but lesser role (Fig 2c). Similarly, total sensitivity indices found that these parameters were the most influential for peak E* production (Fig 2d). The insignificance of [PDE]_*σ*_ for peak E* production indicated that shut-off of R* and E* happened rapidly, before exhausting the population of available E. However, [PDE]_*σ*_ and *k*_*GE*_ were influential for the activation and recovery time courses (Fig 2a and 2b). Their simultaneous occurrence was expected as they appear similarly in the equations for G* and E* production.

Sensitivity indices for the current drop due to flash response, the rms error between simulation and experiment, and the dark current are given in Fig 3. Monte Carlo estimated that the five most influential parameters for goodness of fit were *β*_*dark*_, *a*_*min*_, $J_{ex}^{sat}$, *k*_*R*_, and *k*_*E*_, with each implicated in at least 20% of the variance, and *β*_*dark*_ implicated in as much as 90% (Fig 3c). Moreover, *β*_*dark*_ was also the most influential parameter of the current drop and the dark current (Fig 3a, 3b and 3d). Generally, the total sensitivity indices for the dark current functional exhibited some similarity between its ranking of parameters and those for goodness of fit. This suggested that accurately reproducing the dark current was one of the more important criteria for reproducing experimental results.

Sensitivity indices for the time-to-peak of the current drop and its overshoot are plotted in Fig 4. Monte Carlo estimated that the four most influential parameters for time-to-peak were *k*_*R*_, *k*_*E*_, *k*_*GE*_, and [PDE]_*σ*_ (Fig 4a and 4b). The first two were the rates of shut-off for R* and G*, and their importance was expected. The significance of the second two likely derived from these parameters postponing G* complexing with E*, which in the model was required before G* could decay into inactivated G. Monte Carlo also estimated that an overshoot, where during the flash response the total current temporarily exceeded the dark current value, could not be attributed to any one parameter (Fig 4c). Therefore, an overshoot occurred as a consequence of fundamentally nonlinear interactions between several model parameters. Total sensitivity indices for the overshoot indicated that parameters determining the dark current and time-to-peak were most implicated in its variance (Fig 4d). The uncertainty in these estimates was large, which was partly a consequence of an overshoot occurring infrequently.

## Discussion

The large number of biochemical and geometric parameters together with the relative scarcity of experimental data for cone photoreceptors challenges visual transduction models of increasing biological sophistication. In response, many models in the literature, concerning rods as well as cones, have made simplifying spatial homogeneity assumptions for describing the signaling cascade or aggregated several distinct components of the cascade into simpler phenomenological terms [28, 36, 37, 39, 58–60]. Others have even taken a strictly phenomenological approach to reduce model uncertainty [61]. Faced with such complexity, we retained all the features of our spatio-temporal model [16], and employed a statistical approach to parameter sensitivity analysis that emphasized global behavior (statistical distributions), instead of restricting consideration to local behavior (pointwise derivatives). To better understand the relative importance of parameters across such uncertainty in the literature, we used Sobol indices to quantify which parameters were the most influential for the considered experimental cone flash response of [45]. The advantage of the Sobol method was that it allowed us to encode parameter uncertainty as probability distributions and then decompose the statistical variance of a functional into fractions attributed to any grouping of parameters. In the absence of a compelling reason to do otherwise, we described parameter uncertainty with uniform distributions over simple intervals. While the estimated Sobol indices did depend on the intervals assigned to the parameters, we considered a choice of interval as more robust than a choice of pointwise value. Moreover, this approach could track interactions between parameters while derivative-based sensitivity measures could not.

After reporting known parameter ranges from the literature, these ranges were validated by stochastic optimization, insofar as a parameter set was found that reproduced behaviors of experimental flash responses. The Sobol analysis (Tables 12 and 13 and Figs 2–4) showed the uncertainties in *β*_*dark*_ and *a*_*min*_ to be the most implicated in the error between model prediction and experiment. Among the eight parameters estimated by Monte Carlo to be the most influential for the *L*^2^ error functional, four of these were also influential in determining the dark current as measured by their $S_{i}^{tot}$ indices: *β*_*dark*_ at 94%, *a*_*min*_ at 55%, $J_{ex}^{sat}$ at 30%, and *α*_*max*_ at 15%. The other four could be attributed to shut-off of R*, *k*_*R*_ at 29%, and E*, *k*_*E*_ at 20%, the activation rate of G* by R*, *ν*_*RG*_ at 7%, and the light-induced hydrolysis of cGMP by E*, *k*_*cat*_/*K*_*m*_ at 7%. No single parameter by itself could account for more than an estimated 6% of the model’s error with experiment (Table 12). Consequently, the flash response inextricably depended on nonlinear interactions between model parameters. This conclusion appeared true for the overshoot in the flash response as well. While the uncertainties associated to *J*_*over*_’s $S_{i}^{tot}$ indices were larger than other functionals, which may have been a consequence of an overshoot not occurring in all Monte Carlo trials, the eight parameters estimated as most significant could be loosely characterized as parameters influencing the dark current and parameters influencing the time-to-peak of the flash response. In particular, the value of [PDE]_*σ*_ was shown to be important for time-to-peak but not important for the peak amount of E* produced. In the latter case, this was expected to be a consequence of the rapid shut-off of R* and G* before the available population of E could be exhausted. In the former case, its significance was expected to be a consequence of the model requiring that G* complex with E before it could decay to its inactivated state. In practice, the implications would be that [PDE]_*σ*_ may be most important when it is used to estimate the dark turnover rate of cGMP, *β*_*dark*_. Once that turnover rate is fixed, the significance of [PDE]_*σ*_’s value may be much less. Similar conclusions may hold for the geometric parameters *ν* and *ϵ*_0_, when these are used to estimate a unit conversion between the surface hydrolysis rate of cGMP at the bounding membrane disc and an equivalent volumic rate of cGMP turnover in the thin interdiscal space.

It was not surprising that the geometric parameters *R*_*b*_, *R*_*t*_, *H*, *ν*, and *ϵ*_0_ exhibited relatively little influence on the photoresponse given that the Sobol analysis modeled *β*_*dark*_ as a parameter independent of *ν* and *ϵ*_0_, that the flashes modeled in this work were of uniform intensity throughout the photoreceptor, and that the prescribed uncertainty associated with these parameters was much more narrow than their biochemical counterparts. In [16] it was observed *in silico* that mouse rod biochemistry simply expressed in a fish cone morphology could exhibit a single photon response with an overshoot. In the present work, these parameters were uninvolved in the overshoot for two reasons. First, the ranges of *R*_*b*_ and *R*_*t*_ did not encompass the size of the striped bass cone photoreceptor used in [16]. Second, the single photon response was the most spatially localized case while flashes of uniform intensity were the most homogeneous. We expected that spatial localization created greater opportunity for transduction machinery to become asynchronous as one component may have outpaced another. On the other hand, the parameter *ω*_0_ exhibited a slightly stronger influence on overshoot as indicated by Table 13. This was expected to be a consequence of the sliver angle determining the width across which ion channels were localized on the lateral side.

Our parameter set of Table 10 was broadly consistent with that of other recent modeling efforts. For example [59] found that the dark hydrolysis of cGMP is ∼4x greater in cone than rods and an even higher ratio was given by [36]. Our ratio between *β*_*dark*_ selected in [3] for mouse rod and the value obtained here was ∼ 3x. [59] and [36] reported ∼8–17x faster PDE decay rates in cones compared to rods. In the present work, this rate was ∼13.7x faster. [59] also inferred that the rate of transducin activation by rhodopsin, *ν*_*RG*_, is likely much smaller in cones than rods and an even higher ratio was given by [36]. Although, the value presented in Table 10 was comparable to values reported for mouse rod [3], the obtained rate for activation of PDE* by transducin (*ν*_*GE*_ ≈ 38 *s*^−1^) was much smaller than that derived for mouse rod (*ν*_*GE*_ ≈ 500–1000 *s*^−1^) [8, 62]. Since the current response depended on the E* population, a low value for *ν*_*GE*_ could compensate for *ν*_*RG*_. A somewhat smaller reduction in the activation of PDE* by R* was estimated in red-sensitive cones, but interestingly, not in green-sensitive cones, by [36]. Here and with regard to other parameters, the presently considered model accounted for additional features of the cascade, so some differences compared to other models were to be expected.

The identification of parameters given in Table 10 cannot substitute for future experimental findings and must be refined as more becomes known. A natural future step would be a Bayesian inference of parameter values, and it is expected that such analysis could improve upon the model’s presented experimental predictions. At present, the GSA variance estimates may be strongly influenced by the large uncertainty ranges for some of the parameters, such as *β*_*dark*_ (Tables 8 and 9). This range for *β*_*dark*_ is between 1–2 orders of magnitude larger than that estimated by several other authors. For example, [36] found the dark cGMP turnover rate to be about 10 times faster in cones than rods. As future studies reduce these uncertainties, this approach would be better able to reveal how biological variation in parameters affects the cone responses, e.g., across different types of cones, during light adaptation and in diseased states.

## Conclusion

We end by suggesting, on the basis of Fig 3, the following prioritization of parameters for future measurement and refinement. The turnover rate of cGMP in the dark, *β*_*dark*_, has highest priority. The saturated exchanger current, $J_{ex}^{sat}$, has second highest priority. After these, the rates of R* and E* shutoff, *k*_*R*_ and *k*_*E*_, have priority. While it would certainly be very valuable to quantify *a*_*min*_ directly, this parameter may not be immediately accessible to experiment but instead would have to be estimated from other measurements. This, for example, would be possible in conjunction with Bayesian inference.

## Supporting information

S1 Appendix Supplementary materials. (PDF)
