# Supplementary material for: Phototransduction in retinal cones: Analysis of parameter importance
Source: PLoS One. 2021 Oct 28;16(10):e0258721. doi: 10.1371/journal.pone.0258721 (PMC8553137; doi:10.1371/journal.pone.0258721)
Supplement: S1 Appendix — (PDF) [file pone.0258721.s001.pdf]

A Appendix to *Phototransduction in retinal cones: analysis of parameter importance*

A.1 Dynamics of the cascade

Symbols u and v denote [cG] and $\left[\mathrm{Ca}^{2+}\right]$ respectively. Other symbols are consistent with the main text or are explicitly described in context. These equations are analogous to those used in [1] for dim flashes.

A.1.1 Weak formulation of the dynamics of cGMP

$$\left(\frac{\nu}{1+\nu}\right)\int_{\Omega}\Big(\varphi u_t + D_{cG}\nabla_{\bar{x}}\varphi\nabla_{\bar{x}}u\Big)dx + (\sigma\epsilon_0\cos\gamma_0)\int_S\Big(\varphi u_t + D_{cG}\nabla_S\varphi\nabla_S u\Big)d\sigma$$
$$= \left(\frac{\nu}{1+\nu}\right)\int_{\Omega}\left(\varphi\alpha\left(v\right) - \varphi\frac{k_{\sigma;hyd}}{2}\left([\mathcal{E}]-[\mathcal{E}^*]\right)u - \varphi\frac{k^*_{\sigma;hyd}}{2}\left[\mathcal{E}^*\right]u\right)dx$$

Here $\mathcal{E}$ is the volumic normalization of the surface density E, any one of the two catalytic subunits of PDE, by $\eta = \frac{1}{2}\nu\epsilon_0$. The cyclase synthesis rate is given by

$$\alpha(v) = \alpha_{min} + (\alpha_{max}-\alpha_{min})\frac{K_{cyc}^{m_{cyc}}}{K_{cyc}^{m_{cyc}} + v^{m_{cyc}}}.$$

A.1.2 Weak formulation of the dynamics of Ca^{2+}

$$\left(\frac{\nu}{1+\nu}\right)\int_{\Omega}\Big(\varphi v_t + D_{Ca^{2+}}\nabla_{\bar{x}}\varphi\nabla_{\bar{x}}v\Big)dx + (\sigma\epsilon_0\cos\gamma_0)\int_S\Big(\varphi v_t + D_{Ca^{2+}}\nabla_S\varphi\nabla_S v\Big)d\sigma$$
$$= \int_S\Big(\varphi J_{cG}(u) - \varphi J_{ex}(v)\Big)$$

The CNG channel current and saturated exchanger currents are given by

$$J_{cG}(u) = \frac{f_{Ca^{2+}}}{2B_{Ca^{2+}}\mathcal{F}}\frac{J_{\mathrm{cG}}^{max}}{\Sigma_S}\frac{u^{m_{cG}}}{K_{\mathrm{cG}}^{m_{cG}} + u^{m_{cG}}}$$
$$J_{ex}(v) = \frac{1}{B_{Ca^{2+}}\mathcal{F}}\frac{J_{ex}^{sat}}{\Sigma_S}\frac{v}{K_{ex}+v}$$

Note that Σ_S is the lateral bounding surface area of the *sliver*, the cytoplasmic volume that surrounds the closed section of disks and is encased by plasma membrane, over which the model uniformly distributes ion channels.

A.1.3 Weak formulation of the dynamics for transducer and effector

$$0 = \int_\Omega \left(\varphi \left([\mathrm{R}^*]_\sigma \right)_t + D_R \nabla_{\bar{x}} \varphi \nabla_{\bar{x}} [\mathrm{R}^*]_\sigma + \varphi k_R [\mathrm{R}^*]_\sigma \right) dx$$

$$\begin{aligned} 0 = \int_\Omega \Bigg(& \varphi \left([\mathrm{G}^*]_\sigma \right)_t + D_G \nabla_{\bar{x}} \varphi \nabla_{\bar{x}} [\mathrm{G}^*]_\sigma \\ & - \varphi \nu_{RG} \left(\frac{[\mathrm{G}]_\sigma - [\mathrm{G}^*]_\sigma - [\mathrm{E}^*]_\sigma}{[\mathrm{G}]_\sigma} \right) [\mathrm{R}^*]_\sigma + \varphi k_{GE} \left([\mathrm{E}]_\sigma - [\mathrm{E}^*]_\sigma \right) [\mathrm{G}^*]_\sigma \Bigg) dx \end{aligned}$$

$$0 = \int_\Omega \left(\varphi \left([\mathrm{E}^*]_\sigma \right)_t + D_E \nabla_{\bar{x}} \varphi \nabla_{\bar{x}} [\mathrm{E}^*]_\sigma - \varphi k_{GE} \left([\mathrm{E}]_\sigma - [\mathrm{E}^*]_\sigma \right) [\mathrm{G}^*]_\sigma + \varphi k_E [\mathrm{E}^*]_\sigma \right) dx$$

A.2 Comparison with striped bass flash response

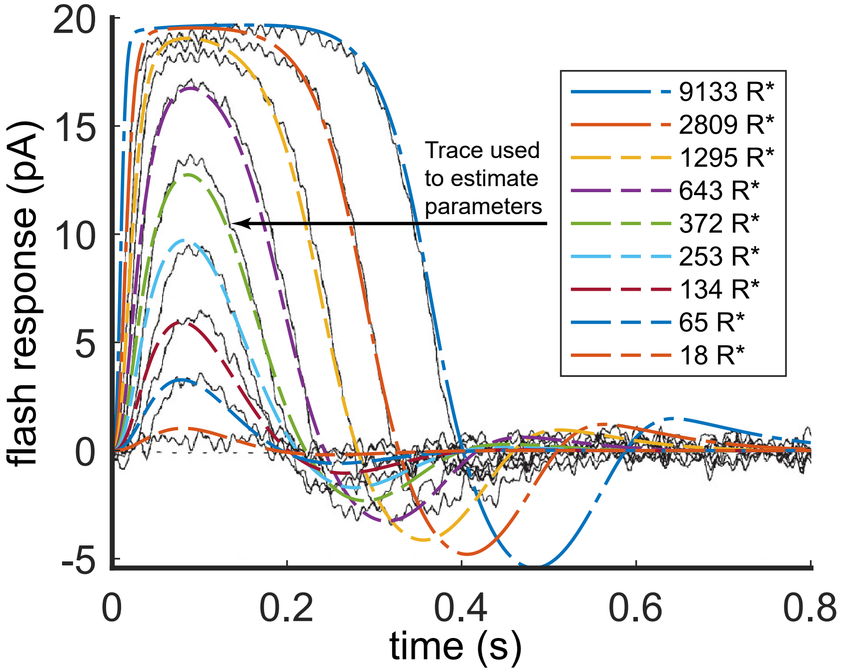

Figure 1: Modeling of flash responses for striped bass cone from [2] with estimated excitations of 18, 37, 91, 178, 372, 913, 1933, 3685, and 9133 isomerizations. Stochastic optimization fit biochemical parameters (Table 1) using the 372 R* flash response (green-dashed line). Other curves were predicted using the same parameter set and listed intensities, which were within a factor 2 of the experimental estimates. With obtained parameters, the model reproduced experimental trends with some deviation in overshoot and flash strength.

Symbol	Units	Definition	Value
α_{max}	$\mu M s^{-1}$	Maximum rate of cGMP synthesis by GC at high $\left[\mathrm{Ca}^{2+}\right]$	**81.3**
$\alpha_{max}/\alpha_{min}$	–	Ratio for GC synthesis of cGMP from low to high $\left[\mathrm{Ca}^{2+}\right]$	**17.3**
β_{dark}	s^{-1}	Rate of cGMP hydrolysis by dark-activated PDE	**7.1**
B_{cG}	–	Buffering power of cytoplasm for cGMP	**1.0**
$B_{Ca^{2+}}$	–	Buffering power of cytoplasm for Ca^{2+}	**26.8**
k_{GE}	$\mu m^2 s^{-1}$	Coupling coefficient for PDE* formation by G*	**0.68**
$[\mathrm{cG}]_{dark}$	μM	Concentration of cGMP in the dark	**3.9**
$\left[\mathrm{Ca}^{2+}\right]_{dark}$	μM	Concentration of Ca^{2+} in the dark	**0.20**
R_b	μm	Radius of COS base	3
R_t	μm	Radius of COS tip	1
ω_0	–	Open margin angle for sliver	π
D_{cG}	$\mu m^2 s^{-1}$	Diffusion coefficient for cGMP	**109.7**
$D_{Ca^{2+}}$	$\mu m^2 s^{-1}$	Diffusion coefficient for Ca^{2+}	15

Continued on next page

Table 1 – *Continued from previous page*

**Symbol**	**Units**	**Definition**	**Value**
D_E	$\mu m^2 s^{-1}$	Diffusion coefficient for PDE*	**1.3**
D_G	$\mu m^2 s^{-1}$	Diffusion coefficient for G*	**1.5**
D_R	$\mu m^2 s^{-1}$	Diffusion coefficient for R*	**1.4**
ϵ_0	nm	Disc thickness	15.0
η	nm	Volume to surface ratio	7.5
$\mathcal{F}$	C/mol	Faraday's constant	96500
$f_{Ca^{2+}}$	–	Fraction of current carried by Ca^{2+}	**0.31**
H	μm	Length of COS	15
J_{dark}	pA	Dark current	**19.76**
J_{cG}^{max}	pA	Maximum CNG channel current (when saturated by cGMP)	**4531**
J_{ex}^{sat}	pA	Saturated exchanger current (when saturated by Ca^{2+})	**4.26**
k_{cat}/K_m	$\mu M^{-1} s^{-1}$	Hydrolytic efficiency of PDE* dimer	**1779**
$k_{\sigma;hyd}$	$\mu m^3 s^{-1}$	Surface hydrolysis rate of cGMP by dark-activated PDE	**5.18e-4**
$k_{\sigma;hyd}^*$	$\mu m^3 s^{-1}$	Surface hydrolysis rate of cGMP by PDE*	**1.48**
k_R	s^{-1}	Rate constant for inactivation of R*	**17.13**
k_E	s^{-1}	Rate constant for inactivation of PDE*	**21.02**
K_{cyc}	nM	Half-saturating $\left[\mathrm{Ca}^{2+}\right]$ for GC activity	**138**
K_{cG}	μM	[cG] for half-maximal CNG channel opening	**25.3**
K_{ex}	μM	$\left[\mathrm{Ca}^{2+}\right]$ for half-maximal exchanger activity	**0.121**
ν	–	Ratio between interdiscal space and disc thickness	1
ν_{ϵ_0}	nm	Interdiscal space thickness	15
ν_{RG}	s^{-1}	Rate of G* formation per R*	**299**
ν_{GE}	s^{-1}	Rate of PDE* formation per G*	**69.5**
n	–	Number of discs	500
N_{Av}	mol^{-1}	Avogadro number	$6.02e23$
m_{cyc}	–	Hill coefficient for GC effect	**2.25**
m_{cG}	–	Hill coefficient for CNG channel	**2.99**
$[\mathrm{G}]_\sigma$	μm^{-2}	Surface density of G	**1320.5**
$[\mathrm{PDE}]_\sigma$	μm^{-2}	Surface density of PDE	**102.8**
σ	–	Ratio between the disc thickness and sliver thickness	1
σ_{ϵ_0}	nm	Distance between the disc rim and outer plasma membrane at sliver	15.0

Table 1: Parameter values found by stochastically minimizing the rms error between experiment and model predictions for a flash intensity of 372 photoisomerizations by the Metropolis-Hastings random walk. Biochemical parameters belong to the same ranges as those used for Sobol analysis in Table 8 of the main text. Geometric parameters were taken from [3].

A.3 Implementing the Metropolis-Hastings algorithm

The Metropolis-Hastings algorithm prescribes a Markov chain whose transitions consist of a proposal probability distribution which generates a candidate and an accept-reject probability distribution that is conditional on the candidate that was proposed. The proposal distribution was designed as a normal random walk over parameter space. Each parameter value was assigned a standard deviation that was 1% of the interval length listed in Table 8. We denote the probability of parameters y being proposed when the chain was currently at parameters x as $p(x; y)$. The accept-reject probability was determined by the choice of proposal distribution $p(x; y)$ and the desired stationary distribution $\pi(x)$. The Metropolis-Hastings algorithm prescribes the probability of accepting candidate y when at x, denoted by $\alpha(x; y)$, as [4, 5]

$$\alpha(x; y) = \min\left(1, \frac{\pi(y)p(y; x)}{\pi(x)p(x; y)}\right)$$

The ratio $p(y; x)/p(x; y)$ was determined by the proposal distribution. From Eq (8) of the main text, the ratio $\pi(y)/\pi(x)$ was determined by the intervals in Table 8, the parameters γ and β, and constraint set K (Table 7) in which all accepted parameter values were required to belong.

The role of γ was to penalize the likelihood of a parameter value being chosen outside of the anticipated range, while β influenced how likely the Markov chain was to pass from a better candidate to a worse candidate for the benefit of space-exploration. The values $\gamma = .6931$ and $\beta = 13.425$ were selected based on empirical performance. The choice of γ ensured that if a parameter was proposed that was twice the length of its interval away from that interval in Table 8, then it was only 25% as likely to be accepted as when it was in the interval. The choice of β ensured that if a proposed parameter set had an rms error that was 1.25 times the current parameter set error, then it was only 5% as likely to be accepted.

A.4 A Summary of the Sobol Method

The Sobol method proceeds as follows: let there be a real-valued functional $f(x_1,\ldots,x_m)$ whose input parameters will be random but independently distributed. Suppose also that, in the joint probability distribution of the parameters, f has a well-defined mean and variance. Then the orthogonal decomposition of f provided by [6] is defined inductively over subsets I of the parameters as

$$\text{For } |I| = 0 : f_0 = E[f]$$

$$\text{For } |I| \leq N : f_{i_1,\ldots,i_k}(x_{i_1},\ldots,x_{i_k}) = E\left[f - \sum_{J \subsetneq I} f_{j_1,\ldots,j_\ell} \mid x_{i_1},\ldots,x_{i_k}\right]$$

The meaning of the $f_{i_1,\ldots,i_k}(x_{i_1},\ldots,x_{i_k})$ is that they are a best projection onto functions which only depend on the smaller collection of parameters $(x_{i_1},\ldots,x_{i_k})$ of what remains of the functional f after lower order interactions between parameters have been subtracted out. This projection is made rigorous through application of the conditional expectation operator, notated by $E[\cdot \mid \cdot]$, in $L^2(\Omega)$. In particular, f_0 is a best projection of the functional f onto constants and so recovers the mean of f. Orthogonality in probability of the $f_{i_1,\ldots,i_k}(x_{i_1},\ldots,x_{i_k})$ follows from the independence of the input parameters. As a consequence,

$$f(x_1,\ldots,x_m) = \sum_{k=0}^{m} \sum_{i_1<\ldots<i_k} f_{i_1,\ldots,i_k}(x_{i_1},\ldots,x_{i_k})$$

$$D = \sum_{k=1}^{m} \sum_{i_1<\ldots<i_k} D_{i_1,\ldots,i_k}$$

where D is the variance of f and $D_{i_1,\ldots,i_m}$ is the variance of $f_{i_1,\ldots,i_k}(x_{i_1},\ldots,x_{i_k})$. The Sobol indices are derived by normalizing the latter sum to 1:

$$S_{i_1,\ldots,i_m} = \frac{D_{i_1,\ldots,i_m}}{D} \tag{1}$$

We continue to follow [6], and for a given subset of parameters y, associate sensitivity and total sensitivity indices

$$S_y = \sum_{k=1}^{m} \sum_{i_1<\ldots<i_k : \forall i_j \in y} S_{i_1,\ldots,i_k} \tag{2}$$

$$S_i^{tot} = \sum_{k=1}^{m} \sum_{i_1<\ldots<i_k : \exists i_j \in y} S_{i_1,\ldots,i_k}. \tag{3}$$

The index S_y measures the percentage of total variance which can be explained by the parameters in y alone while $1 - S_i^{tot}$ measures the percentage of total variance that can be explained using only parameters not in y. Accordingly $S_y \leq S_i^{tot}$. It further follows that $S_y \to 1$ implies f tends to a function only depending on parameters in y, and $S_i^{tot} \to 0$ implies f tends to a function independent of parameters in y. These limits are made precise in the topology of $L^2(\Omega)$.

Computing the indices (Eqs 2-3) relies on their integral expressions [6]. Up to a change of variables, it may be assumed that the input parameters are uniformly distributed over a high-dimensional cube. In this case we are integrating the Lebesgue measure over a unit cube and

$$D_y = \int \int f(y;z)f(y;z')dydzdz' - f_0^2 \tag{4}$$

$$D_y^{tot} = \frac{1}{2} \int \int \left(f(y;z) - f(y';z)\right)^2 dydy'dz. \tag{5}$$

It follows from Eqs 1, 4, and 5 that indices Eqs 2-3 may be directly computed after a Monte Carlo computation of the integrals

$$\int f(x)dx, \quad \int f^2(x)dx, \quad \int \int f(y;z)f(y;z')dydzdz', \quad \int \int \left(f(y;z) - f(y';z)\right)^2 dydy'dz.$$

We follow [7] for efficient computation of the indices S_i, S_{ij}, S_i^{tot}, and S_{ij}^{tot} with respect to the number of model evaluations.

A.5 Confidence intervals for Sobol indices

The Sobol indices were ratios of two Monte Carlo estimated quantities. By the central limit theorem, these Monte Carlo estimates were expected to tend towards a sample from a normal distribution which could be used to construct confidence intervals. In particular, for a normal distribution the population mean was expected to fall within two standard deviations of the sample mean with 95% confidence. It also followed that if the numerator and denominator were incorrectly estimated with at most 5% probability, then their ratio would have had known bounds except for incorrect estimation occurring with at most 10% probability.

These confidence intervals depend on the validity of the normal approximation and that sufficiently many samples have been taken for the central limit theorem to be applicable. Here this assumption was investigated by a bootstrap sampling procedure. (See [8] for example.) The 100,000 Monte Carlo samples were regarded as an empirical approximation to the underlying distribution of the functionals with the parameter ranges of Table 9 in the main text. These were then sampled, independently with replacement, 100,000 times again and the resulting mean was taken. Then this procedure was independently repeated 1,000 times. The resulting 1,000 sample means formed a second empirical distribution which was compared to a normal distribution for verifying central limit theorem behavior. The average error, measured by rms, between the cumulative distribution function (CDF) of these sample means and that of a corresponding normal distribution's CDF, are given in Tables 2-4. Confidence intervals for the Sobol estimates are given in Tables 5-8. Additional convergence spreadsheets are available at [9].

D Conv	E_{act}	E_{peak}	E_{rec}	I_{act}	I_{drop}	T_{peak}	J_{dark}	J_{over}	L^2
rms	0.02	0.01	0.01	0.01	0.01	0.01	0.01	0.01	0.01

Table 2: Average error between the normal distribution's CDF, to which the central limit theorem guaranteed convergence, and the bootstrap sample means' CDF for total functional variance. This average error was measured by the root-mean-square error. These differences were observed to be between 1-2%. The total functional variance formed the denominator of the Sobol indices. The closer the rms error was to 0, the more accurate the confidence intervals for Sobol indices were expected to be.

References

[1] Klaus C, Caruso G, Gurevich VV, DiBenedetto E. Multi-scale, numerical modeling of spatio-temporal signaling in cone phototransduction. PLoS ONE. 2019;14(7):e0219848.

[2] Korenbrot JI. Speed, adaptation, and stability of the response to light in cone photoreceptors: The functional role of Ca-dependent modulation of ligand sensitivity in cGMP-gated ion channels. J Gen Physiol. 2012;139(1):31–56.

[3] Holcman D, Korenbrot JI. Longitudinal diffusion in retinal rod and cone outer segment cytoplasm: the consequence of cell structure. Biophys J. 2004;86:2566–2582.

D_i Conv	E_{act}	E_{peak}	E_{rec}	I_{act}	I_{drop}	T_{peak}	J_{dark}	J_{over}	L^2
rms	**Geometry**								
R_b	0.01	0.01	0.02	0.01	0.01	0.04	0.01	0.01	0.01
R_t	0.01	0.02	0.01	0.02	0.01	0.01	0.02	0.01	0.01
H	0.01	0.01	0.03	0.01	0.02	0.01	0.01	0.01	0.01
ω_0	–	–	–	0.02	0.01	0.02	0.02	0.04	0.01
ϵ_0	0.03	0.01	0.01	0.01	0.03	0.02	0.03	0.02	0.01
ν	0.02	0.01	0.01	0.02	0.04	0.02	0.01	0.03	0.01
σ	–	–	–	0.01	0.07	0.04	–	0.03	0.01
**Catalytic activity and diffusion**									
k_{GE}	0.01	0.02	0.01	0.02	0.01	0.02	–	0.02	0.01
ν_{RG}	0.01	0.01	0.01	0.01	0.01	0.01	–	0.04	0.01
k_R	0.01	0.02	0.01	0.01	0.01	0.01	–	0.03	0.01
k_E	0.01	0.01	0.02	0.01	0.01	0.01	–	0.01	0.01
D_R	0.01	0.02	0.02	0.01	0.01	0.02	–	0.07	0.01
D_G	0.01	0.02	0.01	0.02	0.01	0.01	–	0.02	0.01
D_E	0.02	0.01	0.01	0.03	0.02	0.01	–	0.05	0.01
D_{cG}	–	–	–	0.02	0.02	0.01	–	0.03	0.01
$D_{Ca^{2+}}$	–	–	–	0.03	0.01	0.05	–	0.03	0.03
$[\mathrm{G}]_\sigma$	0.01	0.01	0.02	0.01	0.01	0.01	–	0.02	0.01
**cGMP synthesis and hydrolysis**									
$[\mathrm{PDE}]_\sigma$	0.02	0.02	0.02	0.01	0.01	0.02	0.02	0.03	0.01
β_{dark}	–	–	–	0.01	0.01	0.01	0.01	0.02	0.01
B_{cG}	–	–	–	0.01	0.02	0.02	–	0.02	0.02
k_{cat}/K_m	–	–	–	0.02	0.01	0.01	–	0.04	0.01
α_{max}	–	–	–	0.01	0.01	0.02	0.01	0.01	0.01
a_{min}	–	–	–	0.03	0.02	0.01	0.03	0.02	0.01
m_{cyc}	–	–	–	0.03	0.01	0.01	0.01	0.03	0.02
K_{cyc}	–	–	–	0.01	0.01	0.02	0.01	0.02	0.01
**CNG channel and Ca^{2+} exchanger**									
$B_{Ca^{2+}}$	–	–	–	0.01	0.02	0.02	0.01	0.07	0.01
J_{cG}^{max}	–	–	–	0.02	0.02	0.02	0.01	0.02	0.02
m_{cG}	–	–	–	0.01	0.01	0.01	0.01	0.01	0.01
K_{cG}	–	–	–	0.01	0.02	0.01	0.01	0.06	0.02
$f_{Ca^{2+}}$	–	–	–	0.02	0.01	0.01	0.01	0.04	0.01
J_{ex}^{sat}	–	–	–	0.01	0.01	0.02	0.01	0.01	0.01
K_{ex}	–	–	–	0.02	0.01	0.01	0.01	0.02	0.01

Table 3: Average error between the normal distribution's CDF, to which the central limit theorem guaranteed convergence, and the bootstrap sample means' CDF for single index functional variance. This average error was measured by the root-mean-square error. The single index functional variance formed the numerator of the S_i Sobol indices. The closer the rms error was to 0, the more accurate confidence intervals for Sobol indices were expected to be. The symbol – was used for parameters on which a functional did not depend.

D_i^{tot} Conv	E_{act}	E_{peak}	E_{rec}	I_{act}	I_{drop}	T_{peak}	J_{dark}	J_{over}	L^2
rms	**Geometry**								
R_b	0.01	0.08	0.01	0.01	0.05	0.03	0.01	0.04	0.02
R_t	0.01	0.08	0.01	0.02	0.05	0.02	0.01	0.03	0.03
H	0.01	0.09	0.01	0.03	0.04	0.05	0.01	0.01	0.02
ω_0	–	–	–	0.01	0.01	0.02	0.01	0.02	0.03
ϵ_0	0.01	0.09	0.02	0.02	0.02	0.02	0.05	0.07	0.02
ν	0.01	0.07	0.03	0.03	0.03	0.03	0.10	0.08	0.02
σ	–	–	–	0.02	0.04	0.02	–	0.03	0.03
**Catalytic activity and diffusion**									
k_{GE}	0.03	0.01	0.01	0.01	0.01	0.02	–	0.02	0.03
ν_{RG}	0.01	0.02	0.01	0.01	0.01	0.01	–	0.02	0.01
k_R	0.01	0.02	0.01	0.02	0.01	0.01	–	0.02	0.01
k_E	0.01	0.01	0.01	0.01	0.02	0.01	–	0.01	0.02
D_R	0.01	0.02	0.02	0.02	0.06	0.04	–	0.05	0.03
D_G	0.02	0.02	0.02	0.04	0.04	0.01	–	0.03	0.01
D_E	0.01	0.02	0.01	0.02	0.03	0.02	–	0.06	0.03
D_{cG}	–	–	–	0.01	0.03	0.02	–	0.01	0.03
$D_{Ca^{2+}}$	–	–	–	0.01	0.09	0.06	–	0.01	0.04
$[\mathrm{G}]_\sigma$	0.01	0.08	0.02	0.01	0.02	0.02	–	0.04	0.02
**cGMP synthesis and hydrolysis**									
$[\mathrm{PDE}]_\sigma$	0.01	0.01	0.02	0.01	0.02	0.02	0.05	0.03	0.01
β_{dark}	–	–	–	0.01	0.01	0.01	0.01	0.01	0.01
B_{cG}	–	–	–	0.01	0.02	0.01	–	0.02	0.01
k_{cat}/K_m	–	–	–	0.02	0.01	0.02	–	0.02	0.01
α_{max}	–	–	–	0.04	0.01	0.01	0.01	0.03	0.01
a_{min}	–	–	–	0.02	0.01	0.01	0.01	0.02	0.01
m_{cyc}	–	–	–	0.02	0.02	0.01	0.01	0.08	0.02
K_{cyc}	–	–	–	0.01	0.05	0.02	0.01	0.03	0.02
**CNG channel and Ca^{2+} exchanger**									
$B_{Ca^{2+}}$	–	–	–	0.01	0.03	0.04	0.10	0.02	0.03
J_{cG}^{max}	–	–	–	0.02	0.01	0.02	0.01	0.05	0.02
m_{cG}	–	–	–	0.01	0.02	0.01	0.01	0.02	0.01
K_{cG}	–	–	–	0.01	0.01	0.02	0.03	0.04	0.02
$f_{Ca^{2+}}$	–	–	–	0.01	0.02	0.02	0.01	0.01	0.01
J_{ex}^{sat}	–	–	–	0.02	0.01	0.01	0.01	0.02	0.02
K_{ex}	–	–	–	0.01	0.01	0.01	0.02	0.03	0.01

Table 4: Average error between the normal distribution's CDF, to which the central limit theorem guaranteed convergence, and the bootstrap sample means' CDF for total index functional variance. This average error was measured by the root-mean-square error. The total index functional variance formed the numerator of the S_i^{tot} Sobol indices. The closer the rms error was to 0, the more accurate confidence intervals for Sobol indices were expected to be. The symbol – was used for parameters on which a functional did not depend.

S_i	E_{act}	E_{peak}	E_{rec}	I_{act}	I_{drop}	T_{peak}	J_{dark}	J_{over}	L^2
90% CI	**Geometry**								
R_b	0.00	0.00	0.00	0.00	0.00	0.00	0.00	0.00	0.00
	0.00	0.00	0.00	0.00	0.00	0.00	0.00	0.00	0.00
R_t	0.00	0.00	0.00	0.00	0.00	0.00	0.00	0.00	0.00
	0.00	0.00	0.00	0.00	0.00	0.00	0.00	0.00	0.00
H	0.00	0.00	0.00	0.00	0.00	0.00	0.00	0.00	0.00
	0.00	0.00	0.00	0.00	0.00	0.00	0.00	0.00	0.00
ω_0	0.00	0.00	0.00	0.00	0.00	0.00	0.00	0.00	0.00
	0.00	0.00	0.00	0.00	0.00	0.00	0.00	0.00	0.00
ϵ_0	0.00	0.00	0.00	0.00	0.00	0.00	0.00	0.00	0.00
	0.00	0.00	0.00	0.00	0.00	0.00	0.00	0.00	0.00
ν	0.00	0.00	0.00	0.00	0.00	0.00	0.00	0.00	0.00
	0.00	0.00	0.00	0.00	0.00	0.00	0.00	0.00	0.00
σ	0.00	0.00	0.00	0.00	0.00	0.00	0.00	0.00	0.00
	0.00	0.00	0.00	0.00	0.00	0.00	0.00	0.00	0.00
**Catalytic activity and diffusion**									
k_{GE}	0.24	0.02	0.20	0.00	0.02	0.13	0.00	0.00	-0.01
	0.27	0.02	0.22	0.01	0.02	0.16	0.00	0.00	0.00
ν_{RG}	0.29	0.10	0.00	0.01	0.09	0.00	0.00	-0.01	-0.02
	0.32	0.13	0.00	0.02	0.11	0.00	0.00	0.00	0.01
k_R	0.03	0.24	0.18	0.00	0.13	0.28	0.00	0.00	-0.03
	0.03	0.32	0.20	0.00	0.15	0.34	0.00	0.00	0.03
k_E	0.01	0.14	0.19	0.00	0.08	0.16	0.00	0.00	-0.02
	0.02	0.18	0.21	0.00	0.09	0.20	0.00	0.00	0.03
D_R	0.00	0.00	0.00	0.00	0.00	0.00	0.00	0.00	0.00
	0.00	0.00	0.00	0.00	0.00	0.00	0.00	0.00	0.00
D_G	0.00	0.00	0.00	0.00	0.00	0.00	0.00	0.00	0.00
	0.00	0.00	0.00	0.00	0.00	0.00	0.00	0.00	0.00
D_E	0.00	0.00	0.00	0.00	0.00	0.00	0.00	0.00	0.00
	0.00	0.00	0.00	0.00	0.00	0.00	0.00	0.00	0.00
D_{cG}	0.00	0.00	0.00	0.00	0.00	0.00	0.00	0.00	0.00
	0.00	0.00	0.00	0.00	0.00	0.00	0.00	0.00	0.00
$D_{Ca^{2+}}$	0.00	0.00	0.00	0.00	0.00	0.00	0.00	0.00	0.00
	0.00	0.00	0.00	0.00	0.00	0.00	0.00	0.00	0.00
$[G]_\sigma$	0.00	0.00	0.00	0.00	0.00	0.00	0.00	0.00	0.00
	0.00	0.00	0.00	0.00	0.00	0.00	0.00	0.00	0.00

Table 5: Confidence intervals for the S_i index. Parameters for geometry and catalytic activity and diffusion are shown. Negative numbers were an artifact of the confidence interval construction and may be further simplified to 0.00.

S_i	E_{act}	E_{peak}	E_{rec}	I_{act}	I_{drop}	T_{peak}	J_{dark}	J_{over}	L^2
90% CI	**cGMP synthesis and hydrolysis**								
$[\text{PDE}]_\sigma$	0.20	0.01	0.15	0.00	0.02	0.09	0.00	0.00	0.00
	0.23	0.02	0.17	0.01	0.02	0.12	0.00	0.01	0.01
β_{dark}	0.00	0.00	0.00	0.16	0.26	0.00	0.34	0.00	0.01
	0.00	0.00	0.00	0.24	0.29	0.01	0.41	0.10	0.14
B_{cG}	0.00	0.00	0.00	0.00	0.01	0.00	0.00	0.00	-0.01
	0.00	0.00	0.00	0.00	0.02	0.00	0.00	0.00	0.01
k_{cat}/K_m	0.00	0.00	0.00	0.01	0.09	0.00	0.00	-0.01	-0.02
	0.00	0.00	0.00	0.02	0.11	0.00	0.00	0.00	0.01
α_{max}	0.00	0.00	0.00	0.01	0.00	0.00	0.02	0.00	-0.01
	0.00	0.00	0.00	0.01	0.00	0.00	0.03	0.00	0.02
a_{min}	0.00	0.00	0.00	0.01	0.00	0.00	0.02	0.00	-0.06
	0.00	0.00	0.00	0.02	0.01	0.01	0.03	0.01	0.02
m_{cyc}	0.00	0.00	0.00	0.00	0.00	0.00	0.00	0.00	0.00
	0.00	0.00	0.00	0.00	0.00	0.00	0.00	0.00	0.00
K_{cyc}	0.00	0.00	0.00	0.00	0.00	0.00	0.00	0.00	0.00
	0.00	0.00	0.00	0.00	0.00	0.00	0.00	0.00	0.00
**CNG channel and Ca^{2+} exchanger**									
$B_{Ca^{2+}}$	0.00	0.00	0.00	0.00	0.00	0.00	0.00	0.00	0.00
	0.00	0.00	0.00	0.00	0.00	0.00	0.00	0.01	0.00
J_{cG}^{max}	0.00	0.00	0.00	0.00	0.00	0.00	0.00	0.00	-0.01
	0.00	0.00	0.00	0.00	0.00	0.00	0.00	0.00	0.01
m_{cG}	0.00	0.00	0.00	0.00	0.01	0.00	0.02	0.00	-0.01
	0.00	0.00	0.00	0.00	0.02	0.00	0.03	0.00	0.01
K_{cG}	0.00	0.00	0.00	0.00	0.00	0.00	0.01	0.00	-0.02
	0.00	0.00	0.00	0.00	0.00	0.00	0.02	0.00	0.01
$f_{Ca^{2+}}$	0.00	0.00	0.00	0.00	0.00	0.00	0.00	0.00	-0.02
	0.00	0.00	0.00	0.00	0.00	0.00	0.00	0.00	0.00
J_{ex}^{sat}	0.00	0.00	0.00	0.00	0.00	0.00	0.01	0.00	-0.03
	0.00	0.00	0.00	0.01	0.00	0.00	0.02	0.01	0.04
K_{ex}	0.00	0.00	0.00	0.00	0.00	0.00	0.00	0.00	-0.01
	0.00	0.00	0.00	0.01	0.00	0.00	0.01	0.00	0.02

Table 6: Confidence intervals for the S_i index. Parameters for cGMP synthesis and hydrolysis and CNG channel and Ca^{2+} exchanger are shown. Negative numbers were an artifact of the confidence interval construction and may be further simplified to 0.00.

S_i^{tot}	E_{act}	E_{peak}	E_{rec}	I_{act}	I_{drop}	T_{peak}	J_{dark}	J_{over}	L^2
90% CI	**Geometry**								
R_b	0.00	0.00	0.00	0.00	0.00	0.00	0.00	0.00	0.00
	0.00	0.00	0.00	0.00	0.00	0.01	0.00	0.00	0.00
R_t	0.00	0.00	0.00	0.00	0.00	0.00	0.00	0.00	0.00
	0.00	0.00	0.00	0.00	0.00	0.02	0.00	0.00	0.00
H	0.00	0.00	0.00	0.00	0.00	0.00	0.00	0.00	0.00
	0.00	0.00	0.00	0.00	0.00	0.00	0.00	0.00	0.00
ω_0	0.00	0.00	0.00	0.00	0.00	0.01	0.00	0.03	0.00
	0.00	0.00	0.00	0.00	0.00	0.02	0.00	0.10	0.00
ϵ_0	0.00	0.00	0.00	0.00	0.00	0.00	0.00	0.00	0.00
	0.00	0.00	0.00	0.00	0.00	0.01	0.00	0.00	0.00
ν	0.00	0.00	0.00	0.00	0.00	0.01	0.00	0.00	0.00
	0.00	0.00	0.00	0.00	0.00	0.02	0.00	0.02	0.00
σ	0.00	0.00	0.00	0.00	0.00	0.00	0.00	0.00	0.00
	0.00	0.00	0.00	0.00	0.00	0.01	0.00	0.00	0.00
**Catalytic activity and diffusion**									
k_{GE}	0.35	0.03	0.34	0.14	0.03	0.24	0.00	0.12	0.01
	0.38	0.04	0.36	0.22	0.04	0.29	0.00	0.51	0.01
ν_{RG}	0.42	0.25	0.00	0.13	0.15	0.01	0.00	0.04	0.05
	0.45	0.32	0.00	0.21	0.17	0.02	0.00	0.19	0.10
k_R	0.05	0.57	0.31	0.01	0.21	0.41	0.00	0.08	0.21
	0.06	0.73	0.33	0.01	0.22	0.48	0.00	0.39	0.39
k_E	0.03	0.44	0.34	0.01	0.14	0.25	0.00	0.10	0.15
	0.03	0.58	0.36	0.01	0.15	0.30	0.00	0.53	0.27
D_R	0.00	0.00	0.00	0.00	0.00	0.00	0.00	0.00	0.00
	0.00	0.00	0.00	0.00	0.00	0.00	0.00	0.00	0.00
D_G	0.00	0.00	0.00	0.00	0.00	0.00	0.00	0.00	0.00
	0.00	0.00	0.01	0.00	0.00	0.00	0.00	0.00	0.00
D_E	0.00	0.00	0.00	0.00	0.00	0.00	0.00	0.00	0.00
	0.00	0.00	0.00	0.00	0.00	0.00	0.00	0.00	0.00
D_{cG}	0.00	0.00	0.00	0.00	0.00	0.01	0.00	0.00	0.00
	0.00	0.00	0.00	0.00	0.00	0.03	0.00	0.00	0.00
$D_{Ca^{2+}}$	0.00	0.00	0.00	0.00	0.00	0.00	0.00	0.00	0.00
	0.00	0.00	0.00	0.00	0.00	0.01	0.00	0.01	0.00
$[\mathrm{G}]_\sigma$	0.00	0.00	0.00	0.00	0.00	0.00	0.00	0.00	0.00
	0.00	0.00	0.00	0.00	0.00	0.00	0.00	0.00	0.00

Table 7: Confidence intervals for the S_i^{tot} index. Parameters for geometry and catalytic activity and diffusion are shown.

S_i^{tot}	E_{act}	E_{peak}	E_{rec}	I_{act}	I_{drop}	T_{peak}	J_{dark}	J_{over}	L^2
90% CI	**cGMP synthesis and hydrolysis**								
$[\text{PDE}]_\sigma$	0.31	0.02	0.28	0.12	0.03	0.19	0.00	0.09	0.00
	0.33	0.03	0.30	0.19	0.03	0.24	0.00	0.43	0.01
β_{dark}	0.00	0.00	0.00	0.72	0.38	0.07	0.76	0.41	0.72
	0.00	0.00	0.00	1.04	0.40	0.11	0.89	1.42	1.23
B_{cG}	0.00	0.00	0.00	0.03	0.02	0.02	0.00	0.01	0.01
	0.00	0.00	0.00	0.04	0.03	0.05	0.00	0.04	0.01
k_{cat}/K_m	0.00	0.00	0.00	0.14	0.15	0.03	0.00	0.06	0.05
	0.00	0.00	0.00	0.22	0.17	0.07	0.00	0.36	0.10
α_{max}	0.00	0.00	0.00	0.12	0.02	0.02	0.17	0.02	0.10
	0.00	0.00	0.00	0.21	0.02	0.05	0.20	0.06	0.21
a_{min}	0.00	0.00	0.00	0.24	0.03	0.02	0.28	0.21	0.42
	0.00	0.00	0.00	0.36	0.03	0.04	0.34	0.85	0.74
m_{cyc}	0.00	0.00	0.00	0.00	0.00	0.01	0.00	0.00	0.00
	0.00	0.00	0.00	0.00	0.00	0.04	0.00	0.03	0.00
K_{cyc}	0.00	0.00	0.00	0.00	0.00	0.01	0.00	0.00	0.00
	0.00	0.00	0.00	0.00	0.00	0.04	0.00	0.00	0.00
**CNG channel and Ca^{2+} exchanger**									
$B_{Ca^{2+}}$	0.00	0.00	0.00	0.00	0.00	0.00	0.00	0.07	0.00
	0.00	0.00	0.00	0.00	0.00	0.01	0.00	0.25	0.00
J_{cG}^{max}	0.00	0.00	0.00	0.01	0.00	0.01	0.02	0.00	0.01
	0.00	0.00	0.00	0.02	0.00	0.04	0.03	0.01	0.03
m_{cG}	0.00	0.00	0.00	0.04	0.04	0.03	0.10	0.02	0.03
	0.00	0.00	0.00	0.06	0.04	0.06	0.12	0.08	0.07
K_{cG}	0.00	0.00	0.00	0.05	0.01	0.02	0.08	0.01	0.03
	0.00	0.00	0.00	0.08	0.01	0.04	0.09	0.04	0.07
$f_{Ca^{2+}}$	0.00	0.00	0.00	0.02	0.00	0.02	0.02	0.00	0.03
	0.00	0.00	0.00	0.03	0.00	0.05	0.02	0.00	0.05
J_{ex}^{sat}	0.00	0.00	0.00	0.14	0.00	0.02	0.15	0.28	0.22
	0.00	0.00	0.00	0.25	0.00	0.04	0.19	0.92	0.42
K_{ex}	0.00	0.00	0.00	0.04	0.01	0.02	0.05	0.11	0.04
	0.00	0.00	0.00	0.08	0.01	0.04	0.07	0.38	0.10

Table 8: Confidence intervals for the S_i^{tot} index. Parameters for cGMP synthesis and hydrolysis and CNG channel and Ca^{2+} exchanger are shown. Numbers greater than 1.00 were an artifact of the confidence interval construction and may be further simplified to 1.00.

[4] Brooks S, Gelman A, Jones GL, Meng XL, editors. Handbook of Markov chain Monte Carlo. Chapman & Hall/CRC Handbooks of Modern Statistical Methods. CRC Press, Boca Raton, FL; 2011. doi: 10.1201/b10905.

[5] Robert CP, Casella G. Monte Carlo statistical methods. 2nd ed. Springer Texts in Statistics. Springer-Verlag, New York; 2004. doi: 10.1007/978-1-4757-4145-2.

[6] Sobol IM. Global sensitivity indices for nonlinear mathematical models and their Monte Carlo estimates. Math Comput Simulation. 2001;55(1-3):271–280. doi: 10.1016/S0378-4754(00)00270-6.

[7] Saltelli A. Making best use of model evaluations to compute sensitivity indices. Comput Phys Commun. 2002;145(2):280–297. doi: 10.1016/S0010-4655(02)00280-1.

[8] Kulesa A, Krzywinski M, Blainey P, Altman N. Sampling distributions and the bootstrap. Nature Methods. 2015;12(6):477–478. doi:10.1038/nmeth.3414.

[9] Klaus C. Data for: Phototransduction in retinal cones: Analysis of parameter importance. Dryad, Dataset; 2021. doi:10.5061/dryad.6djh9w11c.
